# Activating Silent Glycolysis Bypasses in *Escherichia coli*

**DOI:** 10.34133/2022/9859643

**Published:** 2022-05-11

**Authors:** Camillo Iacometti, Katharina Marx, Maria Hönick, Viktoria Biletskaia, Helena Schulz-Mirbach, Beau Dronsella, Ari Satanowski, Valérie A. Delmas, Anne Berger, Ivan Dubois, Madeleine Bouzon, Volker Döring, Elad Noor, Arren Bar-Even, Steffen N. Lindner

**Affiliations:** ^1^Max Planck Institute of Molecular Plant Physiology, Am Mühlenberg 1, 14476 Potsdam-Golm, Germany; ^2^Génomique Métabolique, Genoscope, Institut François Jacob, CEA, CNRS, Univ Evry, Université Paris-Saclay, 91057 Evry-Courcouronne, France; ^3^Institute of Molecular Systems Biology, ETH Zürich, Otto-Stern-Weg 3, 8093 Zürich, Switzerland; ^4^Department of Plant and Environmental Sciences, Weizmann Institute of Science, Rehovot, Israel; ^5^Department of Biochemistry, Charité Universitätsmedizin, Virchowweg 6, 10117 Berlin, Germany

## Abstract

All living organisms share similar reactions within their central metabolism to provide precursors for all essential building blocks and reducing power. To identify whether alternative metabolic routes of glycolysis can operate in *E. coli*, we complementarily employed *in silico* design, rational engineering, and adaptive laboratory evolution. First, we used a genome-scale model and identified two potential pathways within the metabolic network of this organism replacing canonical Embden-Meyerhof-Parnas (EMP) glycolysis to convert phosphosugars into organic acids. One of these glycolytic routes proceeds via methylglyoxal and the other via serine biosynthesis and degradation. Then, we implemented both pathways in *E. coli* strains harboring defective EMP glycolysis. Surprisingly, the pathway via methylglyoxal seemed to immediately operate in a triosephosphate isomerase deletion strain cultivated on glycerol. By contrast, in a phosphoglycerate kinase deletion strain, the overexpression of methylglyoxal synthase was necessary to restore growth of the strain. Furthermore, we engineered the “serine shunt” which converts 3-phosphoglycerate via serine biosynthesis and degradation to pyruvate, bypassing an enolase deletion. Finally, to explore which of these alternatives would emerge by natural selection, we performed an adaptive laboratory evolution study using an enolase deletion strain. Our experiments suggest that the evolved mutants use the serine shunt. Our study reveals the flexible repurposing of metabolic pathways to create new metabolite links and rewire central metabolism.

## 1. Introduction

Excluding some marked exceptions (e.g., [1–3]), the central carbon metabolism of all living organisms can be divided into few interacting pathways, i.e., glycolysis, the pentose phosphate pathway, and the tricarboxylic acid (TCA) cycle, each representing highly conserved reaction patterns. The emergence of these conserved patterns can be explained in two complementary ways. First, the structure of central metabolism mostly reflects a metabolic network that could have emerged under primordial conditions where primitive systems prevailed solely by relying on abiotic catalysis [4, 5]. This primordial network could have served as the backbone supporting the emergence of the first cells and, as such, was “frozen” at the origin of life, offering only little flexibility for adaptation throughout the specific evolutionary trajectory of current organisms. Alternatively, and complementarily, central metabolism might constitute an optimal solution for interconverting essential cellular metabolites under a given set of biochemical constraints, including pathway length, favorable thermodynamics, proper kinetics, chemical properties of metabolic intermediates, avoidance of radical enzymes, and toxic intermediates [6–10]. In this scenario, it might be that many pathway solutions have existed which were outcompeted by the ones providing optimal ATP yield (Embden-Meyerhof-Parnas (EMP)) or highest rates (Entner-Doudoroff (ED)).

The universal structure of central metabolism facilitates the study of cellular physiology by enabling to apply knowledge gained from one organism to the understanding of another. On the other hand, this conserved metabolism also severely restricts the metabolic and chemical space we can easily explore and impacts bioproduction by limiting the set of key starting metabolites. Therefore, there is a growing interest in restructuring central metabolism for exploring alternative metabolic networks and pave the way to new bioproduction capabilities, e.g., by replacing EMP glycolysis with the stoichiometrically favorable nonoxidative glycolysis [11–14]. Such novel routes can recruit enzymes from heterologous sources or even integrate new-to-nature metabolic conversions [15]. A possible strategy of this sort is to exploit the native enzymes and pathways of an organism for implementing new pathways that can replace segments of central metabolism. There are two main advantages for this approach. From a practical point of view, it relies only on enzymes that were optimized during evolution to operate within the desired cellular environment. From a scientific perspective, the recruitment of enzymes belonging to different cellular processes to establish novel metabolic networks is akin to the emergence of pathways during evolution and thus provides a platform to explore such key evolutionary events [16].

Here, we followed a systems biology approach of exploring if *Escherichia coli’s* metabolic network contains alternative routes bypassing EMP glycolysis. Engineering these pathways can aid in defining the barriers in which metabolism can operate, providing a better understanding of why glycolysis is shaped in the way it is. Furthermore, we hoped to extend the available design space synthetic metabolism can operate in to tailor metabolism for biotechnological needs. Thus, in this study, we aimed to fully replace the endogenous EMP glycolysis by relying on native enzymes only. An *in silico* analysis identified multiple possible pathways allowing the required conversion of phosphosugars into pyruvate. We engineered two of these routes in *E. coli* strains lacking key EMP glycolysis enzymes. First, we show that a methylglyoxal-dependent route [17–19] can carry all glycolytic flux to support a high growth rate. We also implemented the “serine shunt,” which bypasses glycolysis via serine biosynthesis and deamination to pyruvate. We demonstrate that the operation of this synthetic route relies on a delicate balance between the rate of serine production and consumption, avoiding the inhibitory accumulation of this amino acid. In parallel, we submitted an *E. coli* strain lacking EMP glycolysis to adaptive evolution, which selected for the emergence of the serine shunt after ~140 days of cultivation.

## 2. Methods

### 2.1. Computational Analysis to Identify Glycolytic Bypasses in *E. coli*

In order to identify possible pathways that can bypass EMP glycolysis resorting to native *E. coli* enzymes only, we used a constraint-based approach based on the genome-scale metabolic model of this bacterium (iML1515)) except for a few alterations described in supplementary methods (section titled “Alterations to the iML1515 model”).

This approach is very similar to the one used recently for finding latent carbon fixation cycles in *E. coli* [20]. Namely, we set up a mixed integer linear problem- (MILP-) based optimization problem which simultaneously looks for solutions that balance an objective reaction (here, glycerol to pyruvate) while minimizing the total number of reactions.

The full MILP is formulated as follows:

Minimize

(1)Σi zi.

Such that

(2)vobj=−1,0≤v≤β,z∈0,1n,lnCmin≤x≤lnCmax,0≤−g0−STx+M1−z,v−βz≤0,Sv=0,

where the vector v contains the relative reaction rates, z are the Boolean reaction indicators, x are the log-scaled metabolite concentrations, and $g^{0}$ is a vector of all the reactions’ standard Gibbs free energies in units of *RT*, i.e., ${g_{i}}^{0}=\Delta_{r}G^{\prime 0}\left(i\right)/RT$ (R is the gas constant and T is the temperature in Kelvin). $v_{obj}$ is a new reaction that we add to the iML1515 model with the following chemical formula: $\text{glycerol}+n\;\text{ADP}+n\;{\text{P}}_{\text{i}}⟶\text{pyruvate}+n\;\text{ATP}$ (where n is the required ATP yield). In the first constraint, the rate of the objective reaction ($v_{obj}$) is set to be exactly -1, ensuring that any pathway solution would exactly balance it, and therefore, the overall reaction in the pathway would be glycerol to pyruvate (and the required number of ATPs). All the other constraints are based on the standard thermodynamic flux-balance analysis algorithm [21]. Together, these set of constraints allow only solutions that satisfy mass balance and the second law of thermodynamics, within a given range of flux and metabolite concentration bounds. The parameter β (which we set arbitrarily to 10) sets the upper bound for the rate of each single reaction in the pathway relative to the objective reaction. The lower and upper bounds on the concentrations of most metabolites are set to 1 *μ*M and 10 mM, with the exception of 15 central metabolites and cofactors which are confined to more specific ranges based on physiological data (see Supplementary Table 4).

The objective function (not to be confused with the objective *reaction*) of the MILP is the sum of all $z_{i}$, i.e., the total number of active reactions in the solution. Note that as a preprocessing step, we split all reactions to a forward and backward reaction and therefore all (unidirectional) rates must be positive. The constraint v–βz≤0 ensures that each reaction indicator ($z_{i}$) can be equal to 0, only if the rate is 0. We do not need to care about it being equal to 1 even if a reaction is not active, since the optimization goal (which minimizes the sum of all indicators) will prevent that.

Running the MILP solver once will yield the optimal pathway in terms of overall length (number of activate reactions). In order to explore the space of possible (suboptimal) glycolysis bypasses, we then iterate the solution space using integer cuts to eliminate all the potential solutions within a radius of 2 around each one of the previously found solutions (using the same method as in [20]). We stopped the search after 10 solutions were found (for each possible ATP yield).

### 2.2. Reagents and Chemicals

Primers were synthesized by Eurofins (Ebersberg, Germany) (Supplementary Table S3). Screening PCRs were performed using DreamTaq polymerase (Thermo Fisher Scientific, Dreieich, Germany). PrimeSTAR GXL DNA Polymerase (Takara) was used for gene cloning and amplification of deletion cassettes.

### 2.3. Media

LB medium (1% NaCl, 0.5% yeast extract, and 1% tryptone) was used for molecular biology work and strain maintenance (except *Δeno-* and *Δpgk*-derived strains). When appropriate, kanamycin (25 *μ*g/mL), ampicillin (100 *μ*g/mL), chloramphenicol (30 *μ*g/mL), or streptomycin (100 *μ*g/mL) was used. Medium X (M9+5 g/L casamino acids, 40 mM succinate, and 4 mM glycerol, modified according to [22]) was used for maintenance of glycolysis deletions strains *Δeno* and *Δpgk*. Minimal MA medium (31 mM Na_2_HPO_4_, 25 mM KH_2_PO_4_, 18 mM NH_4_Cl, 1 mM MgSO_4_, 40 *μ*M trisodic nitrilotriacetic acid, 3 *μ*M CaCl_2_, 3 *μ*M FeCl_3_·6H_2_O, 0.3 *μ*M ZnCl_2_, 0.3 *μ*M CuCl_2_·2H_2_O, 0.3 *μ*M CoCl_2_·2H_2_O, 0.3 *μ*M H_3_BO_3_, 1 *μ*M MnCl_2_, 0.3 *μ*M CrCl_3_, 6 H_2_O, 0.3 *μ*M Ni_2_Cl, 6 H_2_O, 0.3 *μ*M Na_2_MoO_4_, 2 H_2_O, 0.3 *μ*M Na_2_SeO_3_, and 5 H_2_O) was used for long-term continuous cultures. For growth analysis, M9 minimal medium was used (50 mM Na_2_HPO_4_, 20 mM KH_2_PO_4_, 1 mM NaCl, 20 mM NH_4_Cl, 2 mM MgSO_4_ and 100 *μ*M CaCl_2_, 134 *μ*M EDTA, 13 *μ*M FeCl_3_·6H_2_O, 6.2 *μ*M ZnCl_2_, 0.76 *μ*M CuCl_2_·2H_2_O, 0.42 *μ*M CoCl_2_·2H_2_O, 1.62 *μ*M H_3_BO_3_, and 0.081 *μ*M MnCl_2_·4H_2_O). The minimal media were supplemented with various carbon sources as indicated in the main text and hereafter.

### 2.4. Strains and Plasmids

*E. coli* strains used in this study were generated from MG1655 derivative strain SIJ488 [23], which was used as wildtype reference (Table 1). The deletions were carried out by *λ*-Red recombineering using kanamycin resistance cassettes generated via PCR using the FRT-PGK-gb2-neo-FRT (Km) cassette (Gene Bridges, Germany) for deletion of *sdaA*, *sdaB*, *tdcB*, *tdcG*, and *mgsA*. For the deletion of *dld*, *gldA*, *gloA*, *aldA, hchA*, and *ppsA*, pKD3 (chloramphenicol) and pKD4 (kanamycin) were used as a template for amplification of deletion cassettes [24]. Primer pairs used are indicated in Supplementary Table S3. Cell preparation and transformation for gene deletion was carried out as described [23, 25]. The coding sequences of the WT sequences of *serA* and the mutated genes were amplified by PCR using the primer pairs serA-pet-F and serA-pet-R (Supplementary Table S3). The amplified fragments were cloned into a modified pET16b expression vector (Table 1) by using In-Fusion cloning kit (Takara, Shiga, Japan). The sequence of the inserts of the resulting plasmids was verified by Sanger sequencing. For exchanging the native promoter of *sdaA* with a constitutive strong promoter, a chloramphenicol resistance cassette was amplified from pKD3 by using the primer pair SdaA-ProEx-F and CAP-sdaA-R. In a second PCR, the promotor sequence (5${}^{\prime }$-ACCTATTGACAATTAAAGGCTAAAATGCTATAATTCCAC-3${}^{\prime }$ [25]) was amplified from pZ-ASS using the primer pair pS-bridge and SdaA-ProEx-R. The purified PCR products were used in a fusion PCR together with the primer pair SdaA-ProEx-F and SdaA-ProEx-R. This resulted in a promoter exchange cassette containing 50 bp flanks to integrate into the intergenic region between *nudL* and *sdaA*. To integrate the feedback-resistant version of 3-phosphoglycerate dehydrogenase (${\text{SerA}}^{*}$, H344A N346A N364A) and simultaneously replace the native promotor of the gene with a strong constitutive one, a chloramphenicol resistance cassette was amplified from pKD3 using primer pair ${\text{serA}}^{*}$-ProEx-F and CAP-${\text{SerA}}^{*}$-R. A PCR product containing a constitutive strong promotor (5${}^{\prime }$-ACCTATTGACAATTAAAGGCTAAAATGCTATAATTCCAC-3${}^{\prime }$ [25]) and the *ser*$A^{*}$ gene was amplified using primer pair pS-bridge and ${\text{serA}}^{*}$-ProEx-R. The purified PCR products were used in a fusion PCR together with the primer pair ${\text{serA}}^{*}$-ProEx-F and ${\text{serA}}^{*}$-ProEx-R, resulting in a chloramphenicol cassette containing *ser*$A^{*}$ behind a strong promotor and a 50 bp flank upstream of the CAT cassette to integrate into the intergenic region behind *rpiA*.

**Table 1:** Strains and plasmids used in this study.

Name	Use/deletion/modification	References
SIJ488	WT	[23]
*Δeno*	*eno* deletion strain	[65]
*Δpgk*	*pgk* deletion strain	[65]
*Δpgk Δeda*	*pgk*, *eda* deletion strain	This study
*Δpgk Δeda Δdld*	*pgk*, *eda*, *dld* deletion strain	This study
*Δpgk Δeda ΔgloA*	*pgk*, *eda*, *gloA* deletion strain	This study
*Δpgk Δeda ΔgldA*	*pgk*, *eda*, *gldA* deletion strain	This study
*Δpgk Δeda ΔaldA*	*pgk*, *eda*, *aldA* deletion strain	This study
*Δpgk Δeda ΔhchA*	*pgk*, *eda*, *hchA* deletion strain	This study
*Δpgk Δeda ΔppsA*	*pgk*, *eda*, *ppsA* deletion strain	This study
*Δtpi*	*tpi* deletion strain	This study
*Δtpi ΔmgsA*	*tpi*, *mgsA* deletion strain	This study
*Δtpi Δdld*	*tpi*, *dld* deletion strain	This study
*Δtpi ΔgloA*	*tpi*, *gloA* deletion strain	This study
*Δtpi ΔgldA*	*tpi*, *gldA* deletion strain	This study
*Δtpi ΔaldA*	*tpi*, *aldA* deletion strain	This study
*Δtpi ΔhchA*	*tpi*, *hchA* deletion strain	This study
*Δtpi ΔppsA*	*tpi*, *ppsA* deletion strain	This study
*Δtpi Δzwf*	*tpi*, *zwf* deletion strain	This study
*Δtpi Δeda*	*tpi*, *eda* deletion strain	This study
*Δ*SerineDA	*sdaA*, *sdaB*, *tdcB*, *tdcG* deletion strain	This study
*Δeno Δ*SerineDA	*eno*, *sdaA*, *sdaB*, *tdcB*, *tdcG* deletion strain	This study
*Δeno Δzwf ΔmgsA* c-*sdaA* c-*ser*$A^{*}$	*eno*, *zwf*, *mgsA* deletion strain, chromosomal overexpression of *ser*$A^{*}$ and *sdaA*	This study
*Δeno Δzwf ΔmgsA* c-*sdaA* c-*ser*$A^{*}$*ΔppsA*	*eno*, *zwf*, *mgsA*, *ppsA* deletion strain, chromosomal overexpression of *ser*$A^{*}$ and *sdaA*	This study
iso1	Glycerol evolved *Δeno* strain	This study
iso1 *ΔmgsA*	*mgsA* deletion in glycerol evolved *Δeno* strain	This study
iso1 *Δzwf*	*zwf* deletion in glycerol evolved *Δeno* strain	This study
iso1 *serA*^WT^	Wildtype reversion of *serA* in glycerol evolved *Δeno* strain	This study
Plasmids		
pZ-ASS	Over-expression plasmid with p15A origin (medium copy number), streptomycin resistance, constitutive strong promoter (5${}^{\prime }$-AATACTTGACATATCACTGTGATTCACATATAATATGCG-3${}^{\prime }$)	[25]
p-*mgsA*	pZ-ASS backbone for overexpression of *mgsA*	This study
p-*ser*$A^{*}$-*serB*-*serC*-*sdaA*	pZ-ASS backbone for overexpression of *ser*$A^{*}$ (H344A N346A N364A), *serB*, *serC*, *sdaA*	This study
pET16b		Novagen
pET16b*-serA*	Overproduction for purification of SerA	This study
pET16b-*ser*$A^{*}$	Overproduction for purification of ${\text{SerA}}^{*}$ (H344A N346A N364A)	This study
pET16b-*serA*(L370M)	Overproduction for purification of SerA (L370M)	This study
pET16b-*serA*(T372N)	Overproduction for purification of SerA (T372N)	This study

### 2.5. Evolution in GM3-Driven Long-Term Continuous Culture

For evolution experiments, precultures of *Δeno* strain were obtained in permissive minimal MA medium supplemented with 20 mM glycerol and 10 mM succinate. The preculture was used to inoculate the growth chambers (16 mL per chamber) of two parallel independent GM3 devices [26]. A continuous gas flow of sterile air through the culture vessel ensured constant aeration and growth in suspension by counteracting cell sedimentation. The cultures were grown in the corresponding medium under turbidostat mode (dilution threshold set to 80% transmittance (OD≈0.4, 37°C) until stable growth of the bacterial population. The cultures were then submitted to a conditional medium swap regime. This regime enabled gradual adaptation of the bacterial populations to grow in a nonpermissive medium which contained 20 mM glycerol only. Dilutions of the growing cultures were triggered every 10 minutes with a fixed volume of medium calculated to impose a generation time of 3.5 hours on the cell population, if not otherwise stated. The growing cultures were fed by permissive or nonpermissive medium depending on the turbidity of the culture with respect to a set OD threshold (OD_600_ value of 0.4). When the OD exceeded the threshold, a pulse of nonpermissive medium was injected; otherwise, a pulse of permissive medium. When the cultures grew on glycerol only (100% nonpermissive medium), the feeding mode was set to turbidostat to increase growth rates. Three isolates were obtained on agar-solidified medium containing glycerol as sole carbon source from both evolution experiments and further analyzed.

### 2.6. Genomic Analysis of Evolved Strains

Pair-end libraries (2×150 bp) were prepared with 1 *μ*g genomic DNA from the evolved strains as well as from the ancestor *Δeno* strain and sequenced using a MiSeq sequencer (Illumina). The PALOMA pipeline, integrated in the platform Microscope (https://mage.genoscope.cns.fr/microscope/home/), was used to map the reads against *E. coli* K12 wildtype strain MG1655 reference sequence (NC_000913.3) for detecting single nucleotide variations, short insertions or deletions (in/dels), and read coverage variations [27]. For genomic analysis of serine-resistant *Δeno* mutants, strains were cultured overnight at 37°C in 4 mL medium X (see Methods, Media). Genomic DNA from overnight cultures was extracted using the NucleoSpin Microbial DNA kit (Macherey-Nagel, Düren, Germany). Construction of libraries for single-nucleotide variant detection and generation of 150 bp paired-end reads on an Illumina Novaseq 6000 platform were performed by Novogene (Cambridge, United Kingdom). Reads were mapped to the reference genome of *E. coli* MG1655 (GenBank accession no. U000913.3). *breseq* pipeline [28] was applied to map the reads against the reference for identification of genomic variants, including SNPs and insertion-deletion polymorphisms (INDELs).

### 2.7. Growth Experiments

4 mL M9 medium containing 10 mM glycerol and 40 mM succinate (permissive growth condition) was used as precultures for growth experiments. Strains were harvested (6,000∗g, 3 min, RT) and washed three times in M9 medium without carbon source. Cultures were inoculated into the M9 media to an OD_600_ of 0.01 in a 96-well microtiter plate (Nunclon Delta Surface, Thermo Scientific). Each well contained 150 *μ*L of culture and 50 *μ*L mineral oil (Sigma-Aldrich) to avoid evaporation. Growth monitoring and incubation at 37°C was carried out in a microplate reader (EPOCH 2, BioTek). In the program, 4 shaking phases of 60 seconds were repeated three times (linear shaking 567 cpm (3 mm), orbital shaking 282 cpm (3 mm), linear shaking 731 cpm (2 mm), and orbital shaking 365 cpm (2 mm)). After the shaking cycles, absorbance at 600 nm was measured. Raw data were converted to 1 cm wide standard cuvette OD values according to $\text{O}{\text{D}}_{\text{cuvette}}=\text{O}{\text{D}}_{\text{plate}}/0.23$. Matlab was used to calculate growth parameters. All experiments were carried out in at least three replicates. Average values were used to generate the growth curves. Variability between triplicate measurements was less than 5% in all cases displayed.

### 2.8. ^13^C Isotopic Labelling Experiments

^13^C-isotope tracing was performed to deduce carbon flux. As described previously [25], 2 mL of an early stationary culture, grown in M9 minimal medium containing 1,6-^13^C_2_-glucose (Sigma-Aldrich, Taufkirchen, Germany) as sole carbon sources, was pelleted and washed in ddH_2_O. The cell pellet was hydrolyzed in 1 mL 6 N HCl at 95°C for 24 h. Subsequently, the HCl was removed by evaporation at 95°C under an air-stream. The hydrolyzed biomass was resuspended in 1 mL ddH_2_O. Amino acid masses were analyzed after separation by ultraperformance liquid chromatography (Acquity, Waters, Milford, MA, USA) using a C18-reversed-phase column (Waters, Eschborn, Germany) as described previously [25, 29]. Mass spectra were acquired by an Exactive mass spectrometer (Thermo Scientific, Dreieich, Germany). Data was analyzed using Xcalibur (Thermo Scientific, Dreieich, Germany). Amino acid standards (Merck, Darmstadt, Germany) were used to determine specific retention times.

### 2.9. RNA Extraction and cDNA Synthesis

Biological triplicates were cultured on M9 minimal medium with either 20 mM glycerol (WT and *Δtpi*, NGR2 iso1) or 4 mM glycerol and 40 mM succinate (WT, *Δtpi*, *Δpgk*) as carbon source. In exponential phase, $\sim 2.5\times 10^{8}$ cells were harvested, mixed with two volumes of RNAprotect™ Bacteria Reagent (Qiagen, Hilden, Germany), pelleted down, and stored at -20°C. Total RNA was extracted using the RNeasy Mini Kit (Qiagen, Hilden, Germany) following the manufacturer’s instructions. In brief, enzymatic lysis was followed by on-column removal of genomic DNA with RNase-free DNase™ (Qiagen, Hilden, Germany) and spin-column-based purification. Integrity and concentration of the isolated RNAs were confirmed by gel electrophoresis and NanoDrop™ One (Supplementary Table S5 A), respectively. Reverse transcription was performed on 500 ng RNA with the qScript™ cDNA Synthesis Kit (QuantaBio, Beverly, MA USA). A RT-negative control has been produced for each sample as reported in the Supplementary Table S5, B. The resulting nucleic acid stock concentration was 25 ng/*μ*L and it was stored at -20°C.

### 2.10. Expression Analysis by Reverse Transcriptase Quantitative PCR (RT-qPCR)

Primers for qPCR have been designed with Eurofins Genomics’ qPCR Assay Design Tool (https://www.eurofinsgenomics.eu/en/ecom/tools/qpcr-assay-design/), based on Prime+ of the GCG Wisconsin Package enhanced with additional parameters for perfect probe design. Primers used in this experiment are shown in Supplementary Table S3. The gene encoding 16S rRNA has been widely used as a bacterial reference gene [30]. It is expressed from *rrsA*, *rrsB*, *rrsC*, *rrsD*, *rrsE*, *rrsG*, and *rrsH* operons [31], and we chose it as well-established transcript for expression normalization of cells in exponential growth. Primer specificity was assessed by conventional PCR on template cDNA using DreamTaq Green™ polymerase (Thermo Fisher Scientific, Dreieich, Germany) (Figure S6). 125 pg of nucleic acid (0.5 *μ*L 1 : 100 stock solution), 1 *μ*M primers (2 *μ*L 2,5 *μ*M primer mix), and 2.5 *μ*L Maxima SYBR™ Green/ROX qPCR Master Mix (Thermo Scientific, Dreieich, Germany) were used per reaction. Three technical replicates were analyzed for each biological replicate. The experiment was carried out with the Applied Biosystems 7900HT Fast Real-Time PCR System (Applied Biosystems) in clear MicroAmp™ Optical 384-well reaction plates with Barcode (Applied Biosystems). Each plate included the respective RNA (RT-) and no-template (NTC) negative controls. Differences in expression levels were calculated according to the 2^-∆∆CT^ method [32–34]. Reported data represents the 2^-∆∆CT^ value that was calculated for each sample individually relative to the average of all biological WT replicate ∆Ct^(Ct(GOI)-Ct(rrsA))^ values. RT-controls showed that residual gDNA contributed to an average of less than 3% of the signal for *mgsA* expression analysis, with a maximum of 7.3%. RT-controls for *serA*, *serB*, *serC*, and *sdaA* expression analysis indicate a wider gDNA contribution to the signal across samples, with an average 8.5%, with one maximum of ~30%. Data shown are not corrected for this gDNA contamination, but gDNA signal is listed in Supplementary Material qPCRs Analysis + RT-controls. Primer efficiency was assessed by qPCR of 6 consecutive tenfold dilutions of 25 ng/*μ*L using wildtype genomic DNA. Efficiency was calculated as $E=\left(10^{\left(-1/\text{slope}\right)}-1\right)\times 100$. The linear range of primers was globally between Cq 9.04 and 27.72. Linear regression showed that primers have approximate efficiencies between 89 and 103.5%, with $R^{2}$ between 0.9988 and 1. The efficiencies of all primers were within the 90–105% range, with the exception of *serB* (89%).

### 2.11. Protein Expression and Purification

The His-taged WT and mutated SerA proteins were expressed in *E. coli* BL21 (DE3) Codon+ (Invitrogen). The cells were grown in 400 mL terrific broth containing 100 *μ*g/mL carbenicillin at 37°C until they reached an $\text{O}{\text{D}}_{600\text{nm}}=2$ upon which expression for 16 h at 20°C was induced by addition 500 *μ*M IPTG. The cells were harvested by centrifugation for 30 min at 10000*g* at 4°C. Cell pellets were frozen at -80°C for one night. Thawed cells were then suspended in 32 mL of Buffer A (50 mM phosphate (Na/K), 500 mM NaCl, 30 mM imidazole, 15% glycerol, and pH 8.0) and lysed for 30 min at room temperature after addition of 3.6 mL of Bug Buster (Novagen), 32 *μ*L DTT (dithiothreitol) 1 M, 320 *μ*L Pefabloc 0.1 M (Millipore), and 23 *μ*L Lysonase (Novagen). Lysate was clarified at 9000*g* for 45 min at 4°C and then loaded onto a 5 mL HisTrap FF column preequilibrated in buffer A. The protein was eluted in buffer B (50 mM phosphate (Na/K), 500 mM NaCl, 250 mM imidazole, 1 mM DTT, 15% glycerol, at pH 8.0) and desalted on a gel-filtration column Hi Load 16/60 Superdex 200 pg in buffer C (50 mM Tris, 50 mM NaCl, glycerol 15%, 1 mM DTT, at pH 8.0). The protein was frozen and stored at -80°C if not immediately used for assays.

### 2.12. Characterization of SerA Kinetic Parameters

Assays were performed using a Safas UV mc2 double beam spectrophotometer at room temperature using quartz cuvettes (0.6 cm path length). Assays of SerA-catalyzed reduction of 2-ketoglutarate were conducted in 40 mM potassium phosphate, 1 mM DTT, at pH 7.5. Kinetic parameters for 2-ketoglutarate were determined by varying its concentration (from 0.005 to 1 mM) in the presence of a saturating concentration of NADH (250 *μ*M). The reactions were monitored by recording the disappearance of NADH at 340 nm (molar extinction $\text{coefficient}=6220\;{\text{M}}^{-1}.\text{c}{\text{m}}^{-1}$). Kinetic constants were determined by nonlinear analysis of initial rates from duplicate experiments using SigmaPlot 9.0 (Systat Software, Inc.).

### 2.13. Characterization of Serine Inhibition of SerA 2-Ketoglutarate Reductase Activity

Standard enzyme assays were conducted with both substrates at saturating concentrations (250 *μ*M NADH and 600 *μ*M 2-ketoglutarate) in 120 *μ*L final volume. Reaction mixes contained 5 *μ*g of purified enzyme. Serine was added at concentrations ranging from 0.5 to 10 *μ*M.

## 3. Results

### 3.1. *In Silico* Analysis of Potential Pathways Bypassing Glycolysis in *E. coli*

**Figure 1:**
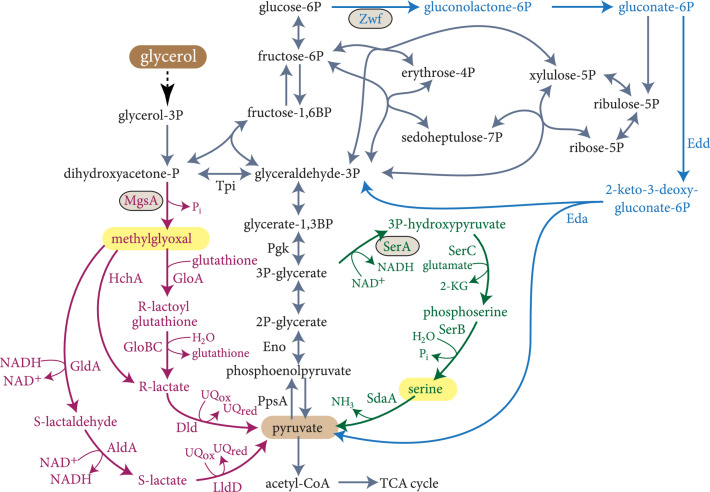
Overview of central metabolism of *E. coli*. Embden-Meyerhof-Parnas (EMP) glycolysis is shown by grey arrows, Entner-Doudoroff pathway is shown by blue arrows, the methylglyoxal bypass is shown in pink, and the serine shunt is shown in green. Key initial committing enzymes of the pathways are circled and namesake intermediates are highlighted in yellow, abbreviated as follows: Entner-Doudoroff pathway: Zwf: glucose 6-phosphate dehydrogenase; Edd: 6-phosphogluconate dehydratase; Eda: 2-keto-3-deoxygluconate 6-phosphate aldolase. Methylglyoxal pathway: MgsA: methylglyoxal synthase; GldA: glycerol dehydrogenase; AldA: aldehyde dehydrogenase; LldD: l-lactate dehydrogenase; HchA: d-lactate dehydratase; GloA: glyoxalase; GloCB: hydroxyacylglutathione hydrolase; Dld: d-lactate dehydrogenase. Serine shunt: SerA: 3-phosphoglycerate dehydrogenase; SerC: phosphoserine aminotransferase; SerB: phosphoserine phosphatase; SdaA: serine deaminase. Glycolysis: Pgk: 3-phosphoglycerate kinase; Eno: enolase; PpsA: PEP synthase.

**Figure 2:**
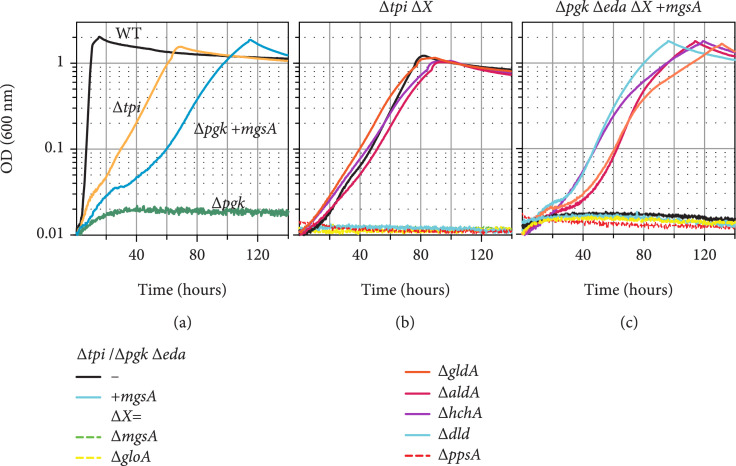
The methylglyoxal pathway bypasses EMP glycolysis in the *Δtpi* strain and *Δpgk Δeda* strain overexpressing *mgsA*. Growth on 20 mM glycerol of *Δtpi* and *Δpgk Δeda +mgsA* strains (a). Growth on 20 mM glycerol of *Δtpi* strain (b) and *Δpgk Δeda* +*mgsA* strain (c) harboring an additional deletion (*ΔX*) of one of the enzymes potentially involved in methylglyoxal degradation. Graphs represent triplicate repeats, showing similar growth (± <5%).

We aimed to uncover latent metabolic routes that can potentially replace the canonical EMP glycolysis and offer new connections between phosphosugar and organic acid metabolism. We used the latest metabolic model of *E. coli* from the BiGG database [35] and systematically searched for all thermodynamically feasible combinations of native reactions that can convert the feedstock glycerol into pyruvate, an organic acid from which all metabolites of the TCA cycle can be derived (Methods). We chose glycerol as the feedstock as it can be directly converted into the simplest phosphosugars—the triose phosphates dihydroxyacetone phosphate (DHAP) and glyceraldehyde 3-phosphate (GAP)—such that its conversion to pyruvate is expected to follow a 1 : 1 stoichiometry. Using this approach, we were able to identify more than 100 thermodynamically feasible routes that can potentially convert glycerol to pyruvate (https://gitlab.com/elad.noor/glycolysis-bypass/-/blob/master/results/figureS2.pdf). These routes and their variants, e.g., routes that share the same intermediates and general metabolic conversions but use different cofactors (e.g., quinones instead of NAD^+^), represent combinations of four broad strategies to convert glycerol to pyruvate (Figure 1): (i) oxidation of triose phosphates to phosphoglycerate, followed by conversion to phosphoenolpyruvate and pyruvate, as within canonical EMP glycolysis; (ii) generation of pyruvate from phosphosugars via the Entner-Doudoroff (ED) pathway (blue arrows in Figure 1); (iii) conversion of triose phosphates to methylglyoxal, which is then oxidized to pyruvate (magenta arrows in Figure 1); and (iv) conversion of 3-phosphoglycerate (3PG) towards serine biosynthesis, followed by serine deamination to pyruvate (green arrows in Figure 1). Since the ED pathway has been analyzed and compared to EMP glycolysis in multiple previous studies [7, 36–38], we decided to focus on the methylglyoxal-dependent and serine-dependent routes and test their potential of bypassing the EMP in *E. coli*. Notably, our approach differs from a previous study which used a similar computational method but included all KEGG database reported reactions (not only from *E. coli*), limiting their search to pathways yielding at least 1 ATP [8]. This method would not find the routes, identified by our approach, via serine or methylglyoxal which are ATP-neutral or even waste ATP, respectively.

### 3.2. A Glycolytic Pathway via Methylglyoxal

*E. coli* is known to channel flux towards methylglyoxal upon accumulation of triose phosphates which results from phosphate depletion limiting the activity of GAP dehydrogenase or from excessive carbon intake [17, 39]. The operation of the methylglyoxal bypass thus enables the cells to adapt to imbalanced central metabolism [40]. Considering the high toxicity of methylglyoxal [41, 42], it was not clear whether the methylglyoxal bypass could indeed support all glycolytic flux without resulting in severe growth defects.

To assess the capability of methylglyoxal metabolism to replace glycolysis, we first constructed a *Δtpi* strain. This strain, when fed with glycerol as sole carbon source, requires the activity of the methylglyoxal shunt to support almost the entire carbon assimilation flux (different to when using glucose as feedstock). To our surprise, when cultivated on glycerol as sole carbon source, the *Δtpi* strain immediately grew without the need for any dedicated overexpression of methylglyoxal synthase (MgsA) or evolution (yellow line in Figure 2(a)). As previously described, a *Δtpi* strain utilizes glucose-derived GAP via EMP and DHAP via the methylglyoxal route [43]. On glycerol, the *tpi* deletion can theoretically be bypassed by the ED pathway. Here, a GAP is “borrowed” to condense with a DHAP to make fructose 1,6-bisphosphate via fructose 1,6-bisphosphate aldolase (Fba) and then use the ED pathway to generate GAP and pyruvate, which both can recover the GAP condensed with DHAP by Fba. In order to exclude this theoretical option, we deleted the genes *zwf* and *eda* individually in the *Δtpi* strain to block the ED pathway. The resulting strains *Δtpi Δzwf* and *Δtpi Δeda* grew similar to the *Δtpi* strain, ensuring no contribution of the ED pathway to the metabolic bypass in the *tpi* deletion strain. An even stronger confirmation of the activity of the methylglyoxal pathway is provided by the fact that the deletion of *mgsA* in the *Δtpi* strain abolished growth (Figure 2(b)). However, one alternative explanation is that the deletion of *mgsA* causes a DHAP accumulation which inhibits growth. We aimed at excluding that this secondary effect, rather than the absence of the methylglyoxal bypass, is responsible for the inability of the *Δtpi ΔmgsA* strain to grow on glycerol. Therefore, we analyzed growth of the Δ*tpi* Δ*mgsA* strain on medium additionally containing succinate. Here, it grew similar to the *Δtpi* strain; thus, toxic effects caused by the absence of *mgsA*, e.g., by DHAP accumulation, can be excluded.

We then constructed a strain lacking EMP glycolysis by deleting phosphoglycerate kinase (*Δpgk*). As expected, the *Δpgk* strain was only able to grow if two carbon sources were provided, feeding both parts of the metabolism (divided by the *pgk* deletion), e.g., glycerol and succinate. As the ED pathway could theoretically bypass the *pgk* deletion, we additionally blocked it by deleting the 2-keto-3-deoxygluconate 6-phosphate aldolase gene (*eda*), resulting in strain *Δpgk Δeda*. In contrast to the *Δtpi* strain, the *Δpgk Δeda* strain required overexpression of the gene coding for methylglyoxal synthase (*mgsA*) from a plasmid to grow on glycerol as sole carbon source (green vs. blue line in Figure 2(a)).

Our results indicate that the methylglyoxal bypass can indeed completely replace glycolysis, despite the high reactivity of its namesake intermediate. Notably, when glycerol is the carbon source, DHAP is expected to accumulate to a high concentration in the *Δtpi* strain, where its metabolism is completely blocked, while in the *Δpgk Δeda* strain such accumulation can be prevented by DHAP conversion to other phosphosugars (Figure 1). As it has been reported that accumulation of DHAP leads to the expression of *mgsA* and activation of the methylglyoxal bypass in *E. coli* [39], we compared the transcription levels of *mgsA* between the WT, *Δtpi*, and *Δpgk Δeda* strains in qPCR experiments on cells grown on M9 minimal medium containing glycerol (WT and *Δtpi*) or glycerol and succinate (WT, *Δtpi*, and *Δpgk Δeda*). When grown on glycerol, *mgsA* transcript levels in *Δtpi* were lower compared to the transcript levels in the WT. Similarly, when glycerol and succinate were the carbon sources, *mgsA* transcript level was lower in the *Δtpi* strain but similar in *Δpgk Δeda* in comparison to the levels determined for the WT (supplementary Figure S2). These results suggest that growth of the *Δtpi* strain fed on glycerol is enabled by an elevated cellular DHAP concentration which enforces a high carbon flux through the methylglyoxal pathway without requiring any change of expression of *mgsA*.

In the case of the *Δtpi* strain fed on glycerol, pyruvate as the final product of the methylglyoxal pathway is needed to supply all parts of central carbon metabolism through gluconeogenesis, TCA cycle, and anaplerotic reactions. Contrarily, in the case of the *Δpgk Δeda* strain overexpressing *mgsA*, pyruvate should be essential for the operation of TCA and anaplerotic reactions and for providing 3-phosphoglycerate as precursor of serine and glycine biosynthesis. Thus, in both strains, a deletion of phosphoenolpyruvate synthase (*ppsA*), which is essential to provide PEP from pyruvate, e.g., anaplerotic reactions, should impede methylglyoxal-dependent growth (Figure 1). Indeed, the *Δtpi ΔppsA* strain and the *Δpgk Δeda ΔppsA* (overexpressing *mgsA*) strain lost the ability to grow on glycerol (Figures 2(b) and 2(c)), thus confirming the pivotal activity of the methylglyoxal bypass.

Methylglyoxal can be converted to pyruvate via three routes (Figure 1, magenta arrows): (i) methylglyoxal attachment to glutathione, followed by hydrolysis to D-lactate and oxidation to pyruvate; (ii) direct electron rearrangement of methylglyoxal to give D-lactate, which is oxidized to pyruvate; (iii) reduction of methylglyoxal to lactaldehyde, followed by oxidation to D-lactate and pyruvate. Previously, the glutathione-dependent pathway was suggested to be the dominant route [19, 40, 44, 45]. However, an unequivocal proof for this claim is missing. To finally settle this question, we used the *Δtpi* and *Δpgk Δeda* strains to generate multiple deletion strains, each carrying the deletion of a different enzyme of the methylglyoxal catabolism. We found that the absence of HchA, GldA, or AldA did not impair growth on glycerol via the methylglyoxal bypass (Figure 2(b)), indicating that the two glutathione-independent routes described above contribute very little, if at all, to methylglyoxal metabolism. On the other hand, the absence of GloA or Dld completely abolished growth on glycerol (Figure 2(b)). These findings confirm the glutathione-dependent route to be indispensable and sufficient for methylglyoxal metabolism. In many cases, ^13^C-labeling experiments are valuable to verify a pathway’s activity, while in the case of the methylglyoxal route, no difference in the labeling pattern compared to EMP glycolysis was expected (Supplementary Fig. S3a); a ^13^C-labeling experiment in the *Δtpi* was carried out using 1,6-^13^C_2_-glucose. The *Δtpi* strain was used because other strains were not able to grow on glucose. 1,6-^13^C_2_-glucose is converted via upper glycolysis to 3-^13^C GAP and 3-^13^C DHAP. In the *Δtpi* strain, GAP is converted to pyruvate via EMP glycolysis and DHAP is catabolized via the methylglyoxal route. As can be seen in Supplementary Fig. S3b, the labeling pattern was similar to the WT and matches the predictions, showing that the ^13^C stays in the C-3 position; thus, no other pathway which involves a carbon rearrangement is active converting the isolated DHAP to pyruvate.

### 3.3. The Serine Shunt Can Replace EMP Glycolysis

The second glycolytic bypass identified by our *in silico* analysis is the serine shunt which combines biosynthesis and degradation of serine (green arrows in Figure 1; https://gitlab.com/elad.noor/glycolysis-bypass/-/blob/master/results/figureS2.pdf). To assess the feasibility of this pathway, we generated an enolase deletion strain (*Δeno*), which cannot operate EMP glycolysis while still being able to generate 3-phosphoglycerate from which the serine biosynthesis pathway begins (Figure 1). For growth on a minimal medium, the *Δeno* strain requires two carbon sources for feeding both upper and lower parts of central carbon metabolism (e.g., glycerol and succinate or xylose and succinate).

We assumed that overexpression of the four enzymes involved in serine biosynthesis from 3-phosphoglycerate and degradation to pyruvate would enable the activity of the serine shunt and support growth of the *Δeno* strain on glycerol only (Figure 1, green arrows). Therefore, we constructed a plasmid constitutively expressing a synthetic operon containing the four following genes: (i) a variant of the native gene coding for 3-phosphoglycerate dehydrogenase which was engineered to remove its native allosteric inhibition by serine (*ser*$A^{*}$, coding for SerA H344A N346A N364A, catalyzing the first step in serine biosynthesis) [46]; (ii) *serC*, coding for phosphoserine aminotransferase; (iii) *serB*, coding for phosphoserine phosphatase; and (iv) *sdaA*, coding for the major serine deaminase isozyme in *E. coli*, converting serine into pyruvate [47]. However, the resulting plasmid (p-*ser*$A^{*}$-*serB*-*serC*-*sdaA*) failed to support growth of the *Δeno* strain on glycerol. Moreover, PCR analysis of the transformants revealed that the strains did not carry the whole operon but only fractions of it. We assume that this occurred due to toxic effects of the overexpression of the serine biosynthesis genes and hence resulted in gene inactivation/removal from the plasmid.

**Figure 3:**
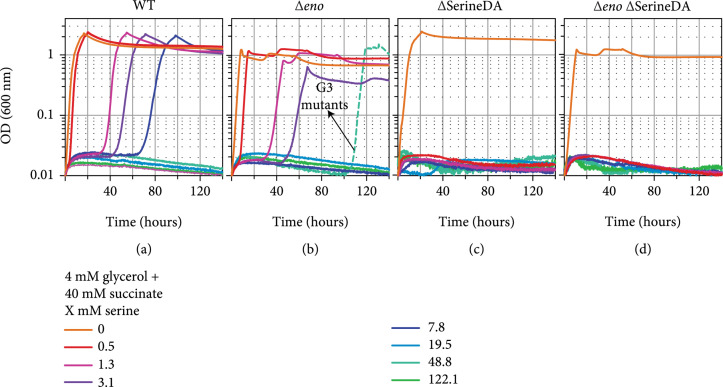
Serine inhibits growth at low millimolar concentrations in WT *E. coli* and abolishes growth at submillimolar concentrations in strains lacking serine deaminase (“SerineDA”) enzymes. Strain WT (a), *Δeno* (b), *Δ*SerineDA (c) (*ΔsdaA ΔsdaB ΔtdcB ΔtdcG*), and *Δeno Δ*SerineDA (d) (*Δeno ΔsdaA ΔsdaB ΔtdcB ΔtdcG*) were incubated in media containing 4 mM glycerol and 40 mM succinate. Serine concentrations were added as indicated. Three isolates (G3#1, #2, and #3) were obtained from the *Δeno* culture growing in the presence of 48.8 mM serine after an extended lag-phase and analyzed by genome sequencing. Graphs represent triplicate repeats, showing similar growth (±5%).

**Figure 4:**
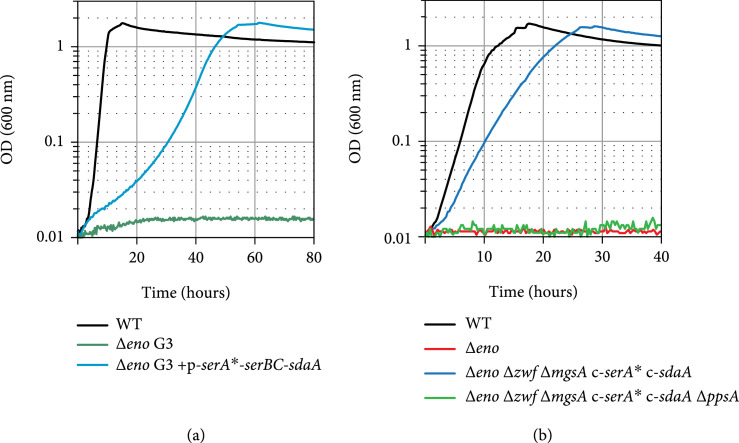
Growth on glycerol of serine-resistant *Δeno* isolate G3#1 transformed with p-*ser*$A^{*}$-*serB*-*serC*-*sdaA* (a) and *Δeno* strain with chromosome-integrated *ser*$A^{*}$ and *sdaA* genes (b) *Δeno* c-*sdaA* c-*ser*$A^{*}$). Growth experiments were performed in at least three repeats, showing similar growth behavior (±5%).

Serine’s toxicity is well known and can be attributed, at least partially, to its deamination to the highly reactive and toxic compound hydroxypyruvate [48]. Indeed, we found that addition of even small amounts of serine (1-8 mM) substantially delayed the growth of the WT (Figure 3(a)) and the *Δeno* strain (Figure 3(b)) with glycerol and succinate as co-carbon sources. The *Δeno* strain seems to be more sensitive to serine than the WT strain, as its growth was completely inhibited at serine concentrations of ~8 mM. Further deletion of the genes coding for serine deaminases (*ΔsdaA ΔsdaB ΔtdcB ΔtdcG* [49]), thus removing serine sinks, increased the sensitivity of both the WT and the *Δeno* strain further: even small amounts of serine (0.5 mM) completely inhibited growth on glycerol and succinate (Figures 3(c) and 3(d)). Interestingly, in one of the *Δeno* strain cultures incubated in the presence of 48.8 mM serine, the cells began to grow after >100 hours (Figure 3(b)). This suggests the emergence of mutations enabling the cells to tolerate this high level of serine. We isolated three individual serine-tolerant strains (G3 mutants) and sequenced their genomes. In the resulting reads, we found sequence differences in *pcnB* encoding poly(A) polymerase I and *leuB* encoding 3-isopropylmalate dehydrogenase of leucine biosynthesis, both not directly connectable to the serine insensitivity of the mutants. But, in all three isolated strains, a somewhat different genomic region was multiplied several fold (as indicated by a 4-8 fold increased sequencing coverage, Supplementary Table S1 and Supplementary Fig. S4): 1,761,810 bp to 1,816,965 bp (G3 #3), 1,762,676 bp to 1,781,465 bp (G3 #1), or 1,762,962 bp to 1,798,478 bp (G3 #2). Notably, these amplified genomic regions harbor the genes *sufA* and *sufB*, coding for subunits of the iron-sulfur cluster scaffold complex [50]. Among its other functions, this cluster serves as an essential component of the primary serine deaminase enzyme SdaA [51]. Hence, it seems likely that increasing the availability of iron-sulfur clusters in the cell contributed to a faster and more efficient degradation of serine, making the cells more tolerant to this amino acid. To test if the increased tolerance to serine would enable the mutated strain to grow on glycerol via the serine shunt, we overexpressed the four genes described above. Indeed, we found that transformation of the G3 mutant strains with p-*ser*$A^{*}$-*serB*-*serC*-*sdaA* enabled them to grow on glycerol as sole carbon source (Figure 4(a)). It therefore seems that the above-mentioned changes supported a strong metabolic sink for toxic serine to enable high flux via serine biosynthesis and degradation with minimal adverse effects.

In order to decrease and stabilize the rate of serine biosynthesis to avoid its accumulation, we pursued a strategy of gene expression from the chromosome rather than from a plasmid in the naïve *Δeno* strain. The genes *ser*$A^{*}$ and *sdaA* were overexpressed from the chromosome (see method section), while the native expression was preserved for the genes *serB* and *serC*. We found that overexpression of *ser*$A^{*}$ from the chromosome in the *Δeno* strain is possible only after the chromosomal overexpression of *sdaA* is established, again pointing to the necessity of a balanced serine synthesis and degradation activity for growth via the serine shunt (data not shown). Finally, to avoid any carbon flux through the methylglyoxal bypass and the ED pathway, we deleted *mgsA* and the gene encoding glucose 6-phosphate dehydrogenase (*Δzwf*). The *Δeno ΔmgsA Δzwf* strain overexpressing *ser*$A^{*}$ and *sdaA* from the chromosome was indeed able to grow on glycerol with no need for prior adaptation (Figure 4(b)). As expected, the deletion of *ppsA*, which blocks the conversion of pyruvate to phosphoenolpyruvate, abolished growth (Figure 4(b)), thus confirming that this essential metabolite cannot be produced by some variant of EMP glycolysis that bypasses or replaces the enolase reaction. Overall, our results indicate that the serine shunt can be implemented in the cells to replace the canonical EMP glycolysis using a rational engineering approach, where the rate of serine biosynthesis is balanced with that of serine degradation, consequently avoiding deleterious accumulation of this amino acid.

### 3.4. Continuous Culture Evolution Results in the Emergence of the Serine Shunt

As an alternative option to the rational engineering of a synthetic glycolysis bypass, we resorted to continuous culture protocols to investigate which of the three bypass routes—ED pathway, methylglyoxal bypass, or serine shunt—would emerge naturally during long-term cultivation of the *Δeno* strain under selective conditions (we chose to start with the *Δeno* strain as it has the potential to activate all three mentioned bypass routes). We applied a medium-swap regime using GM3 continuous cultivation devices [26, 52] to a growing population of the *Δeno* strain, fed alternatively with a permissive medium containing both glycerol and succinate, and a nonpermissive medium containing only glycerol (Methods). When the turbidity of the culture, measured in real time, was below a predefined value, the culture was diluted with the permissive medium; otherwise, the nonpermissive medium was used to dilute the culture. Such a medium-swap protocol enables gradual adaptation of the bacterial population to conditions it initially cannot grow in [26, 52], in our case proliferation without succinate as substrate of the lower part of the carbon metabolism.

**Figure 5:**
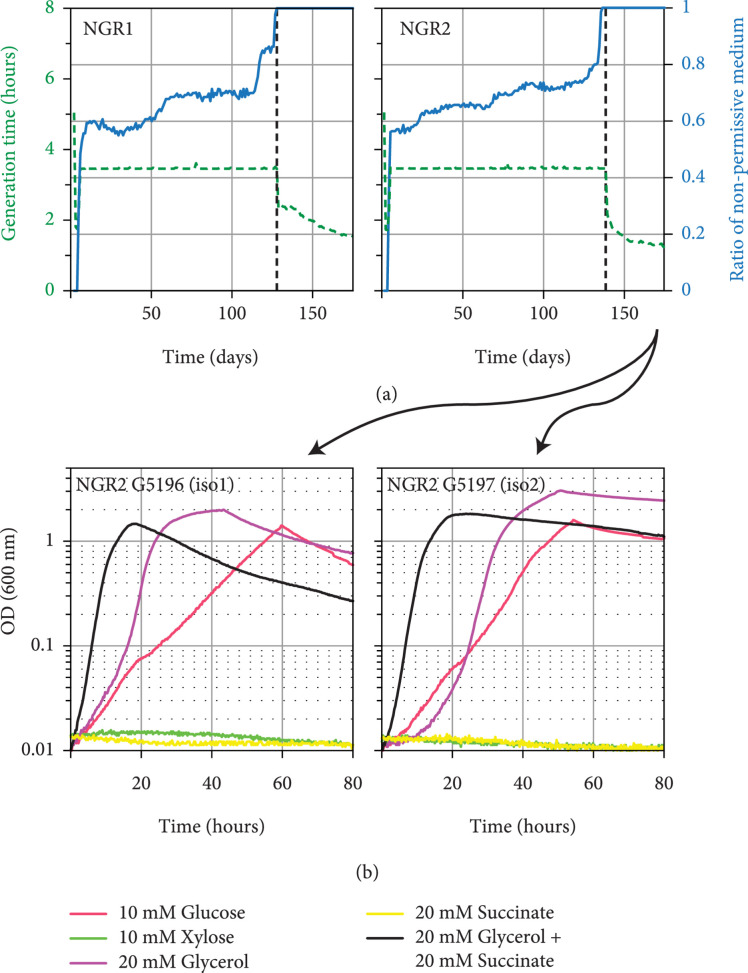
Adaptive evolution in continuous culture of the *Δeno* strain to growth on glycerol. (a) Two independent cultures were subjected to a medium swap regime in GM3 devices (see Methods). Blue lines show the ratio of nonpermissive medium over permissive medium (right axis). The nonpermissive medium contained 20 mM glycerol, and permissive medium contained 20 mM glycerol plus 10 mM succinate. The generation time of the growing population was set to 3.5 hours. Once steady growth was obtained on glycerol as sole carbon source, the bacterial population was cultivated under turbidostat regime. Generation times are indicated by the green dashed lines (left axes). (b) Growth analysis of two isolates from NGR2 on the indicated carbon sources. In all cases, growth experiments were performed in triplicate, showing similar growth (±5%).

We conducted the adaptive evolution experiment in two parallel cultures, both of which adapted to grow on glycerol as sole carbon source after 130-140 days amounting to more than 900 generations (Figure 5(a)). We then applied a turbidostat mode—cultivating the cells solely on glycerol and diluting the culture every time a predefined turbidity is reached—to evolve the culture towards a higher growth rate (Figure 5(a)). We isolated two strains from each of the evolved cultures and sequenced their genomes. Strains isolated from each of the evolved cultures showed highly similar mutation profiles (Supplementary Table S2). All sequenced strains harbored a nonsynonymous mutation in *serA*, either replacing T372 with asparagine or replacing L370 with methionine. No mutation was observed in genes coding for enzymes participating in the methylglyoxal shunt or the ED pathway. This suggested that the adaptive evolution of the *Δeno* strain led to the emergence of the serine shunt, rather than to any other of the possible glycolytic bypass routes. However, neither known bypasses (ED and MG) nor other latent bypasses can be excluded at this point. We found that the isolates from only one of the evolved cultures could stably grow on glycerol alone when cultivated within a 96-well plate (Figure 5(b)). The strain iso1 was further characterized.

To exclude that an unknown pathway which involves carbon rearrangement is active in the iso1 strain, ^13^C-labeling experiments were performed. The iso1 strain was grown in 1,6-^13^C_2_-glucose which is converted via upper glycolysis to 2 molecules of 3-^13^C-GAP which then is converted to 3-^13^C-phosphoglycerate. Regardless which route is taken, EMP, ED, methylglyoxal, or serine shunt, the labeling pattern in amino acids derived from respective metabolites should be identical (Supplementary Fig. S3) unless a pathway involving carbon rearrangement is active. Indeed, the labeling pattern of the iso1 strain was identical to the labeling of the WT control in all amino acids analyzed (Supplementary Fig. S3). Thus, carbon rearrangement can be excluded.

**Figure 6:**
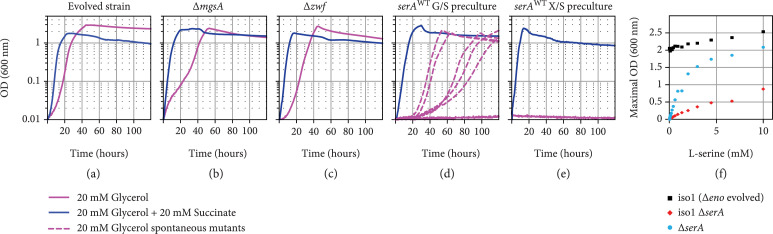
Growth of an evolved *Δeno* strain (iso1) and derivatives deleted in key enzymes of glycolysis bypassing routes demonstrate the activity of the serine shunt. Growth of the iso1 strain (a), with an additional deletion in *mgsA* (b), *zwf* (c), or reversion of the mutated *serA* (L370M) into the wildtype version (d and e). Cultures of *serA*^WT^ were inoculated from precultures growing in glycerol+succinate (d) or xylose+succinate (e). Growth was recorded with glycerol (pink line) and glycerol+succinate (blue line). Comparison of maximal OD (600 nm) of iso1 *ΔserA* and a *ΔserA* strain on 20 mM glycerol supplemented with a serine gradient (Supplementary Fig. S7 for growth experiment with serine gradient) (f). Growth of at least 2 independent biological replicates (8 in the case of *serA*^WT^ reversion) was analyzed in three technical repeats, showing similar growth behavior (±5%).

The four reactions of the serine shunt usually carry much less flux than needed for the serine shunt to operate as sole glycolytic option. To analyze if the expression of the corresponding genes is altered in the iso1 strain, we performed qPCR experiments after growth on glycerol. The transcript level of *serC* and *serB* genes was increased in the iso1 strain compared to the WT reference, showing a 11- and 2.5-fold increased RNA level of *serC* and *serB*, respectively. Surprisingly, *serA* and *sdaA* expressions did not significantly differ from the WT control (Supplementary Fig. S5). To ensure that no glycerol is converted via the alternative bypasses, we deleted their key reactions in the iso1 strain. Neither deletion of *mgsA* nor *zwf* abolished growth (Figures 6(a)–6(c)), indicating that the glycolysis bypass in the iso1 strain does not rely on methylglyoxal bypass or the ED pathway. Despite our continuous efforts, we were unable to delete *sdaA*, which suggests that serine deamination is essential for growth or survival also in rich medium (e.g., Medium X, see Methods), pointing again to the serine shunt as the bypassing route.

To further analyze the operation of the serine shunt in the evolved strain, we restored the WT version of the gene *serA* in strain iso1 and performed growth tests on glycerol. The recombinant SerA WT derivatives of iso1 had lost the ability to grow on glycerol, but the growth phenotype appeared very leaky. Interestingly, we observed contrasting growth behaviors between various experimental replicates (Figure 6(d)): some were unable to use glycerol as sole carbon source, and others started to grow slowly after a short lag-phase, while a few grew faster after a similar delay. These observations strongly indicated the emergence of mutations. Indeed, Sanger sequencing of PCR-products of the *serA* locus of every isolate from a growing culture revealed that SerA was mutated. Each of the tested strains contained one of the following mutations: A367T, H342Y, L332P, or R338C, while none of them harbored the SerA variants L370M or T372N identified in isolates from the long-term evolution experiments. Since these mutants arose rather quickly, within hours or days, we concluded that they already appeared in the precultures, which contained glycerol and succinate. Replacing glycerol with xylose for alimenting the upper part of central metabolism in the preculture prevented the emergence of mutants growing on glycerol as sole carbon source within the duration of the experiment (>5 days) (Figure 6(e)).

Finally, we deleted the *serA* gene in the iso1 strain. A *serA* deletion blocks serine biosynthesis and, hence, turns the iso1 strain into a serine auxotroph. This strain allowed us to test the serine demand of the strain and compare it to a WT deleted in *serA* (*ΔserA*). If a different pathway than the route via serine biosynthesis would be used, the serine demand of both strains, iso1 *ΔserA* and *ΔserA*, would be similar. But, if growth of the glycolytic bypass active in the iso1 strain is dependent on the serine shunt, the supplemented serine would account for all biomass produced from lower metabolism, compared to serine derived biomass building blocks in the *ΔserA* strain only. As expected, both strains, iso1 *ΔserA* and *ΔserA*, did not grow without serine supplementation, and, when a gradient of serine was supplemented to the medium containing 20 mM glycerol, the iso1 *ΔserA* strain required much higher serine concentrations to reach similar ODs compared to the *ΔserA* strain (Figure 6(f), Supplementary Fig. 7). These results indicate that the majority of the serine provided to the iso1 *ΔserA* strain is used for providing carbon to feed lower metabolism, e.g., TCA cycle intermediates.

### 3.5. Mutated SerA Variants Lost Feedback Inhibition by Serine

The observations described above point to the pivotal role of the SerA reaction for the activity of the serine biosynthesis and degradation pathway as glycolytic bypass: e.g., inability to delete the serine deaminase gene *sdaA* in the evolved strains, mutations fixed in *serA* during the evolution experiment, and spontaneous mutations arising in *serA* in the evolved genetic context of iso1 strain where the WT allele of *serA* had been restored. Interestingly, all the mutations identified in SerA in the course of our study are located in the C-terminal allosteric regulatory domain of the enzyme and thus could possibly modulate the negative allosteric feedback of L-serine on SerA activity. To test whether this hypothesis was correct, we characterized both the kinetic parameters and the regulatory effect of L-serine on enzyme activity of the purified SerA WT, the SerA variants which emerged during the evolution experiments (SerA T372N, culture NGR1) and (SerA L370M, culture NGR2), as well as the triple mutation SerA variant (H344A N346A N364A), which was used for the rational engineering of the serine shunt and was previously reported to be feedback-resistant [46]. The promiscuous 2-ketoglutarate reductase activity of SerA was monitored, which, in contrast to the oxidation of 3-phosphoglycerate, is a thermodynamically favorable reaction and is known to be also regulated by L-serine [53]. All the variants tested exhibited 2-ketoglutarate reductase activities comparable with that of the WT enzyme (Table 2), which is in accordance with the fact that none of the mutations were close to the catalytic site of the enzyme [46]. As expected, the activity of WT SerA was strongly decreased in the presence of micromolar concentrations of L-serine (0.5-10 *μ*M) (Table 2). This concentration range corresponds to the previously published IC_50_ for l-serine, which was determined to be between 2 and 10 *μ*M [53]. By contrast, the SerA variants showed a significantly lower sensitivity to the presence of L-serine, as demonstrated by the minimal decrease of the activity of L370M, T372N, and H344A N346A N364A SerA variants with increasing l-serine concentration (Table 2). Our findings in both, the isolated mutants as well as in the rationally engineered strains, support that the operation of the serine shunt is highly dependent on the presence of a feedback-resistant SerA variant. Kinetic properties of the downstream serine deaminase SdaA (*K_M_* of ~2.7 mM for l-serine) support this conclusion. The *K_M_* is three orders of magnitude higher than the IC_50_ of SerA for l-serine [51]; thus, a feedback-inhibited SerA variant might not allow sufficient flux via the pathway to achieve l-serine concentrations high enough for SdaA activity.

**Table 2:** Apparent steady-state kinetic parameters of SerA WT and variants and inhibition by serine.

Enzyme	Km^a^ (mM)	kcat^a^ (s^-1^)	kcat/Km (M^-1^ s^-1^)	Inhibition by serine (*μ*M)^b^					
				0	0.5	1	2	5	10
SerA WT	0.033	0.346	$1.04\times 10^{4}$	100	104	63	40	17	7
SerA H344A N346A N364A	0.007	0.483	$6.80\times 10^{4}$	100	92	89	89	96	94
SerA T372N (NGR1)	0.012	0.364	$2.93\times 10^{4}$	100	89	83	81	79	82
SerA L370M (NGR2)	0.019	0.359	$1.88\times 10^{4}$	100	97	99	107	94	54

^a^Apparent steady-state kinetics of SerA variants measured with 2-ketoglutarate. ^b^2-Ketoglutarate reductase activity of SerA variants was measured under saturation conditions in the presence of the indicated *μ*M l-serine concentrations. Data was normalized to 100% enzyme activity without l-serine addition.

## 4. Discussion

Our computational analysis revealed the presence of multiple feasible glycolytic bypasses in *E. coli*’s native metabolic network. All of these were combinations of the canonical catabolic EMP and ED pathways and two cryptic routes, which so far had not been described as a significant bypass to EMP glycolysis in any organism (in the case of the serine shunt) or were only described as a sink for excess carbon (i.e., the methylglyoxal route) [18, 19, 43]. Here, we were able to engineer these two glycolytic alternatives in *E. coli*, relying exclusively on native enzymes. Previous studies observed the emergence of the methylglyoxal pathway in a *Δtpi* strain during growth on glucose, thus splitting carbohydrate metabolism between EMP glycolysis from GAP and methylglyoxal metabolism from DHAP [18], and the serine shunt was proposed to improve acetyl-CoA precursor supply for the bioproduction of poly(3-hydroxybutyrate) [54]. But as of yet, to the best of our knowledge, both pathways have not been described as a complete glycolytic bypass.

In addition to the rational engineering of the pathways, a directed evolution experiment evolving an enolase deletion strain was conducted, leaving all bypass options open for natural selection. This experiment resulted in the emergence of the serine shunt. Intuitively, a bypass via the ED pathway, which operates in *E. coli* when growing on gluconate [55], might seem more likely to occur. But, evolving the ED pathway to bypass the glycolytic blockade presumably requires the accumulation of several adaptive mutations susceptible to increase the gluconeogenic flux, to inactivate/downregulate the 6-phosphogluconate dehydrogenase and to enhance phosphogluconate dehydratase (*edd*) and 2-keto-3-deoxygluconate 6-phosphate aldolase (*eda*) activity. In contrast, and in line with the outcome of the evolution experiment, engineering the functional serine shunt required only the balanced overexpression of a feedback-resistant SerA variant and of SdaA. Curiously, we showed that the methylglyoxal pathway needed only the overexpression of MgsA in the *Δpgk* strain; therefore, it was expected that the methylglyoxal pathway could be established most easily as a glycolytic bypass by evolution. One reason for the selection of the serine shunt instead of the methylglyoxal pathway during evolution of the enolase deletion strain might be the higher intracellular concentration of 3-phosphoglycerate (~4 mM) compared to DHAP (~0.5 mM) during growth on glycerol [56]. The 3-phosphoglycerate concentration, determined for WT *E. coli*, is likely even higher in the *Δeno* strain; this together with the lower ATP cost of the serine shunt might have triggered the fixation of mutations establishing the serine shunt as *Δeno* bypass. Noteworthy, in a previous study, a pyruvate auxotrophic strain was evolved for the emergence of pyruvate generating reactions; here, the underlying pathway remained unclear but computational analysis revealed several pathway options, among them also the serine and the methylglyoxal option [57].

As expected, reverting the *serA* mutation which emerged in the evolved *Δeno* strain abolished growth on glycerol as sole carbon source. However, after a short period of incubation, growth associated with the appearance of various point mutations in the *serA* gene was restored. This indicates that the strain underwent adaptation processes during the evolution, possibly priming its metabolism for the use of the serine shunt. These can also be seen in the qPCR results of the involved genes. However, these adaptations are not directly reflected in any of the mutations identified in the genome sequencing. A further indication of the activity of the serine shunt was provided by the strain’s high demand of serine supplementation to the medium, when serA was deleted in the evolved strain (iso1 *ΔserA*). When compared to a WT *ΔserA* reference, significantly higher serine concentrations were needed by iso1 *ΔserA* in order to reach similar OD (600 nm). All together our experiments provide strong evidence for the activity of the serine shunt in iso1.

Both of the glycolytic bypasses contain toxic intermediates. In our experiments, only the toxic effects of L-serine caused a stress response in the strain transformed with the plasmid overexpressing serine biosynthesis and degradation genes. On the other hand, no additional overexpression of methylglyoxal degradation enzymes was necessary to implement the methylglyoxal bypass, indicating the sufficiently fast native glutathione-dependent conversion of the highly reactive methylglyoxal to D-lactate. Besides involving a toxic intermediate, the serine shunt presents a thermodynamic barrier at the level of 3-phosphoglycerate dehydrogenase, which additionally is highly inhibited by low concentrations of L-serine.

While many reactions of central metabolism can be catalyzed using different electron acceptors, e.g., PQQ-dependent glucose oxidation [58], or cosubstrates, e.g., PPi instead of ATP for phosphorylations by phosphofructokinase or pyruvate-phosphate dikinase [59], the general structure of central metabolism remains unchanged. Why some glycolytic pathways and their variants emerged and manifested throughout all organisms to convert sugars into biomass building blocks, while other biochemical possibilities either never appeared in nature or were discarded during evolution, is not well understood. Some insights came from previous studies of the connection between sugar catabolism and the organism’s environment (i.e., anaerobic or aerobic). There is evidence that ATP yield outweighs protein cost of a pathway when operating under anaerobic conditions; hence, the EMP pathway is dominantly used under these conditions. Contrarily, obligate aerobic organisms prefer the ED pathway, which ensures a lower protein cost and a higher thermodynamic driving force. Accordingly, facultative anaerobic organisms like *E. coli* possess both pathway options [7]. The methylglyoxal pathway and serine shunt, besides their structural differences, significantly differ in their ATP balance, i.e., consumption of ATP (methylglyoxal pathway) versus no consumption/formation of ATP (serine shunt) per pyruvate generated. These differences in ATP yield render both pathways infeasible in the absence of terminal electron acceptors, e.g., under fermentative conditions. This might provide an explanation for the absence of these pathways in nature. However, the nonphosphorylative ED pathway operates as the route for glucose to pyruvate conversion in some archaea and hence similar to the serine shunt produces no net ATP yield [60].

In the past decade, important breakthroughs were achieved in the field of synthetic metabolism. The implementation of C1 assimilatory routes for the fixation of CO_2_ [61, 62], formate [63], or formaldehyde [64], enabling biomass production from renewable resources, are examples of audacious attempts to rewire central carbon metabolism. While primarily motivated by the need to provide sustainable options for the industrial production of fuels and chemicals, these studies accelerate the understanding of the underlying principles of metabolism. Other examples like the implementation of phosphoketolase-dependent nonoxidative glycolysis [12, 13] illustrate the pace synthetic biology has adopted to define the limits and barriers which might have shaped the structures of central metabolism at the onset of cellular life. Our approach, combining rational design of nonnatural pathways and experimental evolution, is in line with this effort trying to “make sense of the biochemical logic of metabolism” [6] and at the same time opens opportunities for new bioproduction routes of potential industrial relevance, e.g., for optimizing production of serine, cysteine, or 1,2-propanediol.

## Data Availability

The data supporting the presented findings are available within the paper or in its Supplementary files. Strains and plasmids used here are available on request from the corresponding author. Data from the following public repositories were used in this study: BiGG (http://bigg.ucsd.edu/), KEGG (https://www.kegg.jp/), and eQuilibrator (http://equilibrator.weizmann.ac.il/).

## Additional Points

*Code Availability*. The code used in this study can be found at Zenodo (https://zenodo.org/record/6509926).

## Supplementary Materials

Supplementary Materials Supplementary Figures in GitLab: computationally identified EMP bypasses. Figure S1: growth of a *Δ*tpi *Δ**mgsA* strain on glycerol and succinate compared to a *Δ**tpi* strain. Figure S2: *mgsA* transcript levels determined by qPCR experiments. Figure S3: predicted and measured ^13^C-labeling in selected amino acids upon feeding of 1,6-^13^C_2_-glucose in cells using EMP-glycolysis, the methylglyoxal pathway, or the serine shunt. Figure S4: genome sequencing coverage of serine-tolerant *Δ**eno* isolates (G3 mutants). Figure S5: transcript level of serine shunt genes of the glycerol evolved iso1 strain. Figure S6: target specificity analysis of qPCR primers. Figure S7: serine-dependent growth of iso1 *Δ**serA* strain compared to a WT-based *Δ**serA* strain. Table S1: identified mutations different in the serine-tolerant *Δ**eno* strains compared to the reference strain. Table S2: identified mutations in the evolved *Δ* eno strains. Table S3: oligonucleotide primers used. Supplementary Method to the computational analysis to identify glycolytic bypasses in *E. coli*/A constraint-based method for finding glycolysis bypasses. Table S4. Allowed metabolite concentration ranges in the model. Table S5: RNA samples and reverse transcription information.
