# Supplementary material for: Activating Silent Glycolysis Bypasses in Escherichia coli
Source: Biodes Res. 2022 May 11;2022:9859643. doi: 10.34133/2022/9859643 (PMC10521649; doi:10.34133/2022/9859643)
Supplement: Supplementary Materials — Supplementary Figures in GitLab: computationally identified EMP bypasses. Figure S1: growth of a Δtpi ΔmgsA strain on glycerol and succinate compared to a Δtpi strain. Figure S2: mgsA transcript levels determined by qPCR experiments. Figure S3: predicted and measured 13C-labeling in selected amino acids upon feeding of 1,6-13C2-glucose in cells using EMP-glycolysis, the methylglyoxal pathway, or the serine shunt. Figure S4: genome sequencing coverage of serine-tolerant Δeno isolates (G3 mutants). Figure S5: transcript level of serine shunt genes of the glycerol evolved iso1 strain. Figure S6: target specificity analysis of qPCR primers. Figure S7: serine-dependent growth of iso1 ΔserA strain compared to a WT-based ΔserA strain. Table S1: identified mutations different in the serine-tolerant Δeno strains compared to the reference strain. Table S2: identified mutations in the evolved Δ eno strains. Table S3: oligonucleotide primers used. Supplementary Method to the computational analysis to identify glycolytic bypasses in E. coli/A constraint-based method for finding glycolysis bypasses. Table S4. Allowed metabolite concentration ranges in the model. Table S5: RNA samples and reverse transcription information. [file 9859643.f1.zip › 220501_Supplementary.docx]

**Supplementary information to**

**Activating silent glycolysis bypasses in *Escherichia coli***

Camillo Iacometti^1,#^, Katharina Marx^1,#^, Maria Hönick^1^, Viktoria Biletskaia^1^, Helena Schulz-Mirbach^1^, Ari Satanowski^1^, Beau Dronsella^1^, Valérie A. Delmas^2^, Anne Berger^2^, Ivan Dubois^2^, Madeleine Bouzon^2^, Volker Döring^2^, Elad Noor^3,4^, Arren Bar-Even^1^, Steffen N. Lindner^1,5,^*

^1^ Max Planck Institute of Molecular Plant Physiology, Am Mühlenberg 1, 14476 Potsdam-Golm, Germany.

^2^ Génomique Métabolique, Genoscope, Institut François Jacob, CEA, CNRS, Univ Evry, Université Paris-Saclay, 91057 Evry-Courcouronne, France.

^3^ Institute of Molecular Systems Biology, ETH Zürich, Otto-Stern-Weg 3, 8093 Zürich, Switzerland.

^4^ Department of Plant and Environmental Sciences, Weizmann Institute of Science, Rehovot, Israel.

^5^ Department of Biochemistry, Charité Universitätsmedizin, Virchowweg 6, 10117 Berlin, Germany.

**Supplementary Figures in** [**GitLab**](https://gitlab.com/elad.noor/glycolysis-bypass/-/blob/master/results/figureS2.pdf): *In silico* identified thermodynamically feasible glycolytic routes which could potentially convert glycerol to pyruvate. For the identification the latest metabolic model of *E. coli* from the BiGG database (*1*) was used.

**Supplementary Figure S1**: Growth of Δ*tpi and* Δ*tpi* Δ*mgsA* on M9 medium containing 20 mM glycerol and 20 mM succinate. Growth experiments were performed in at least three repeats, showing similar growth behavior (± 5%).

**Supplementary Figure S2**: Relative transcript levels of *mgsA*. Cells were harvested in exponential phase (OD600 0.5-0.6) from M9 minimal medium cultures. For comparison of WT to Δ*tpi* 20 mM glycerol was used as a carbon source, for comparison of WT, Δ*tpi* and Δ*pgk* 4 mM glycerol and 40 mM succinate were used as a carbon source. mRNA levels of *mgsA* was determined by RT-qPCR as described in the methods section and normalized to transcript level of 16S rRNA (*rrsA*). Data significance: *, p < 0.05, **, p < 0.01, ***, p < 0.005.

**Supplementary Figure S3**: Expected labeling distribution upon feeding of 1,6-^13^C_2_-glucose in strains using glycolysis via EMP, methylglyoxal, or the serine shunt (a). ^13^C-labeling experimentally found in 3-phosphoglycerate and pyruvate derived amino acids (b). Data represent the means of three independent replicates with errors < 5 %.

**Supplementary Figure S4**: **Genome sequencing coverage in the serine tolerant strain as analyzed by *breseq*.** After sequencing the genomes of the three isolated strains (Δ*eno* G3 #1,2,3) and their ancestor strain (Δ*eno*), the results were mapped against the *E. coli* MG1655 reference genome (GenBank accession no. U000913.3). The graphs indicate the coverage of the reads against the genome position for the whole length (left) and the read increased area (right). *Unique total* (blue lines) refers to reads with only one best match to the reference genome; *repeat total* (red lines) refers to reads having multiple equally good matches to repeat sequences; *total* (green line) is the sum of both *unique* and repeat *matches.*

**Supplementary Figure S5**: Relative transcript levels of *serA, serB, serC,* and *sdaA* in iso1 and WT strains. Cells were harvested in exponential phase (OD_600_ 0.5-0.6) after growth on M9 minimal medium containing 20 mM glycerol. mRNA levels of *serA, serB, serC,* and *sdaA* were determined by RT-qPCR as described in the methods section and normalized to the transcript level of 16S rRNA (*rrsA*). Data significance: *, p < 0.05, **, p < 0.005.

**Supplementary Figure S6: Target specificity of qPCR primers. (A)** Table reporting amplicon length (*Amp. bp*) and target gene’s genomic coordinates. 16S rRNA primers target six of the seven rRNA operons. **(B)** 2% agarose gel picture confirms the products’ size amplified from genomic DNA using the qPCR primers.

**Supplementary Figure S7**: Growth of iso1 Δ*serA* and a Δ*serA* strain on 20 mM glycerol supplemented with a serine gradient. Data represents growth of at least 2 independent biological replicates, analyzed in three technical repeats with similar growth behavior (± 5%).

**Supplementary Table S1: Mutations identified in the serine tolerant Δ*eno* strains.** The genomes of three isolates growing with 48.8 mM serine (Δ*eno* G3 #1,2,3) and their ancestor strain (Δ*eno*) were sequenced. X indicates the presence of the mutation (compared to the *E. coli* MG1655 reference genome (GenBank accession no. U000913.3) in the respective sequencing result.

| **position** | **mutation** | **annotation** | **gene** | **∆*eno* G3 #1** | **∆*eno* G3 #2** | **∆*eno* G3 #3** | **∆*eno*** |
| --- | --- | --- | --- | --- | --- | --- | --- |
| 81,261 | A→T | L233Q (CTG→CAG) | *leuB* ← |  |  |  | **X** |
| 158,823 | Δ4 bp | coding (301‑304/1398 nt) | *pcnB* ← | **X** |  |  |  |
| 158,845 | (AGGCGGCAGTTACGGAACAGT)_1→2_ | coding (282/1398 nt) | *pcnB* ← |  |  |  | **X** |
| 158,883 | C→A | D82Y (GAC→TAC) | *pcnB* ← |  | **X** |  |  |
| 257,908 | Δ776 bp |  | *[crl]* | **X** | **X** | **X** | **X** |
| 1,405,877 | T→C | intergenic (+228/‑102) | *abgR* → / → *smrA* | **X** | **X** | **X** | **X** |
| 1,978,503 | Δ776 bp |  | *insB1*–*insA* | **X** | **X** | **X** | **X** |
| 1,979,486 | IS*5* (+) +4 bp | intergenic (‑271/‑264) | *insA* ← / → *uspC* | **X** | **X** | **X** | **X** |
| 2,173,365 | Δ2 bp | pseudogene (913‑914/914 nt) | *gatC* ← | **X** | **X** | **X** | **X** |
| 3,560,455 | +G | intergenic (‑2/+1) | *glpR* ← / ← *glpR* | **X** | **X** | **X** | **X** |
| 4,296,381 | +GC | intergenic (+587/+55) | *gltP* → / ← *yjcO* | **X** | **X** | **X** | **X** |
|  | | | | | | | |
| **Unassigned new junction evidence** | | | | | | | |
| **position** |  |  | **gene** |  |  |  |  |
| = 70387 |  |  | *araC* | **X** | **X** | **X** | **X** |
| 4171751 = |  |  | *rrfB* |  |  |  |  |
| 1207790 = |  | coding (290/630 nt) | *stfP* |  | **X** |  |  |
| 1209619 = |  | pseudogene (1/501 nt) | *stfE* |  |  |  |  |
| = 1299498 |  | coding (305/630 nt) | *stfP* |  | **X** |  |  |
| 1300698 = |  | pseudogene (18/501 nt) | *stfE* |  |  |  |  |
| = 1299498 |  | intergenic (+253/‑1684) | *ychE/oppA* | **X** | **X** |  |  |
| 1300698 = |  | intergenic (+1453/‑484) | *ychE/oppA* |  |  |  |  |
| 1761810 = |  | coding (703/747 nt) | *sufC* |  |  | 1061 (4.000) |  |
| = 1816965 |  | coding (171/750 nt) | *chbG* |  |  |  |  |
| 1762676 = |  | coding (1334/1488 nt) | *sufB* | 680 (3.340) |  |  |  |
| = 1781465 |  | coding (71/1290 nt) | *ydiS* |  |  |  |  |
| 1762962 = |  | coding (1048/1488 nt) | *sufB* |  | 1718 (8.160) |  |  |
| = 1798478 |  | coding (465/984 nt) | *pheS* |  |  |  |  |
| 3911839 = |  | coding (1830/1830 nt) | *glmS* | **X** | **X** | **X** |  |
| = 4171849 |  | intergenic (+93/‑208) | *rrfB/murB* |  |  |  |  |

**Supplementary Table S2:** **Identified mutations in the evolved Δ*eno* strains.**

| **NGR1** | **chromosomal position*** | **mutation type** | **mutation event** | ***mutated gene*** | **intragenic position** | **amino acid change** | **intergenic mutation (distance to the flanking genes)** | **amplified / deleted region** | **mutations identified in the indicated strain (•)** | |
| --- | --- | --- | --- | --- | --- | --- | --- | --- | --- | --- |
|  |  |  |  |  |  |  |  |  | **G5181** | **G5182** |
|  | 4114812 | A/T | SNP | *glpK* | 434 | I145N |  |  | • | • |
|  | 2098629 | A/T | SNP | *gnd* | 664 | W222R |  |  | • | • |
|  | 1913509 | A/T | SNP | *proQ* | 50 | L17Q |  |  | • | • |
|  | 3813831 | A/C | SNP |  |  |  | *pyrE/rph(40/55)* |  | • | • |
|  | 4183596-604 |  | DEL 9 bp | *rpoC* | 224-232 |  |  |  | • | • |
|  | 3055318 | G/T | SNP | *serA* | 1115 | T372N |  |  | • | • |
|  |  |  |  |  |  |  |  | DEL(*gntU-yhhY*) | • | • |
|  |  |  |  |  |  |  |  | DEL(*cpxP*) | • | • |
|  |  |  |  |  |  |  |  | x2 *(zur-insA*) | • | • |
|  |  |  |  |  |  |  |  |  |  |  |
| **NGR2** | **chromosomal position*** | **mutation type** | **mutation event** | ***mutated gene*** | **intragenic position** | **amino acid change** | **intergenic mutation (distance to the flanking genes)** | **amplified / deleted region** | **mutations identified in the indicated strain (•)** | |
|  |  |  |  |  |  |  |  |  | **G5196 (iso1)** | **G5197 (iso2)**  **(is** |
|  | 2973447 | C/T | SNP | *aas* | 590 | R197Q |  |  | • | • |
|  | 2058550 | G/A | SNP | *cbl* | 389 | T130M |  |  | • |  |
|  | 2291468 | T/C | SNP | *ccmF* | 1459 | M487V |  |  | • | • |
|  | 2534052 | G/A | SNP | *crr* | 197 | G66D |  |  | • | • |
|  | 2872235 | G/A | SNP | *cysN* | 1207 | P403S |  |  | • |  |
|  | 1250341 | C/T | SNP | *dhaR* | 53 | T18I |  |  | • |  |
|  | 3533277 | A/T | SNP | *envZ* | 614 | L205Q |  |  | • | • |
|  | 2460903 | -/C | INS |  |  |  | *fadL/yfdF* (235/131) |  | • |  |
|  | 1140280 | T/- | DEL |  |  |  | *flgL/rne* (71/125) |  | • | • |
|  | 1975804 | A/T | SNP | *flhC* | 65 | L22Q |  |  | • | • |
|  | 379237 | G/- | DEL |  |  |  | *frmR/frmRAB (132/56)* |  | • | • |
|  | 2940307 | C/- | DEL | *gcvA* | 283 |  |  |  | • | • |
|  | 3048203 | C/T | SNP | *gcvT* | 487 | A163T |  |  | • |  |
|  | 4115926 | G/T | SNP | *glpF* | 188 | S63Y |  |  | • | • |
|  | 3090583 | -/G | INS | *gshB* | 684 |  |  |  | • | • |
|  | 2600510 | T/C | SNP | *hyfB* | 671 | M224T |  |  | • |  |
|  | 2601551 | G/A | SNP | *hyfB* | 1712 | G571D |  |  | • | • |
|  | 722302 | A/G | SNP | *kdpD* | 1336 | W446R |  |  | • | • |
|  | 3476916 | C/T | SNP | *kefB* | 1714 | A572T |  |  | • |  |
|  | 673763 | A/G | SNP | *leuS* | 244 | F82L |  |  | • | • |
|  | 1937431 | G/A | SNP | *lpxM* | 787 | R263C |  |  |  | • |
|  | 2155124 | G/A | SNP | *mdtB* | 1838 | G613D |  |  | • | • |
|  | 2157817 | G/A | SNP | *mdtC* | 1408 | A470T |  |  |  | • |
|  | 819024 | A/G | SNP |  |  |  | *moaE/ybhL* (54/83) |  | • | • |
|  | 979114 | C/T | SNP | *mukB* | 3566 | A1189V |  |  | • | • |
|  | 2857276 | C/A | SNP | *mutS* | 2162 | A721E |  |  | • | • |
|  | 1283205 | C/T | SNP | *narH* | 379 | R127C |  |  | • | • |
|  | 1534614 | -/A | INS | *narV* | 28 |  |  |  |  | • |
|  | 1913362 | G/T | SNP | *proQ* | 197 | S66* |  |  | • | • |
|  | 1368060 | T/- | DEL |  |  |  | *pspE/ycjM* ((33/180) |  | • | • |
|  | 3928170 | A/T | SNP | *ravA* | 947 | L316H |  |  | • | • |
|  | 1413508 | C/T | SNP | *recE* | 1903 | A635T |  |  |  | • |
|  | 4180066 | C/T | SNP | *rpoB* | 799 | R267C |  |  | • | • |
|  | 1708175 | T/C | SNP | *rsxD* | 1010 | L337P |  |  | • | • |
|  | 3055325 | G/T | SNP | *serA* | 1108 | L370M |  |  | • | • |
|  | 560505 | C/T | SNP | *sfmD* | 1586 | S529L |  |  |  | • |
|  | 3105232 | G/A | SNP | *speC* | 1946 | A649V |  |  | • | • |
|  | 3259260 | T/C | SNP | *tdcE* | 1181 | Q394R |  |  | • | • |
|  | 3078126 | T/C | SNP | *tktA* | 1532 | D511G |  |  | • |  |
|  | 3177089 | G/A | SNP | *tolC* | 953 | G318E |  |  | • | • |
|  | 1329648 | G/A | SNP | *topA* | 577 | A193T |  |  |  | • |
|  | 1331254 | A/G | SNP | *topA* | 2183 | Y728C |  |  | • |  |
|  | 1058654 | -/G | INS | *torA* | 176 |  |  |  | • |  |
|  | 1491778 | A/T | SNP | *trg* | 1285 | I429F |  |  |  | • |
|  | 3606830 | C/T | SNP | *tusA* | 190 | V64I |  |  | • |  |
|  | 131055 | A/G | SNP | *yacH* | 206 | V69A |  |  | • |  |
|  | 248170 | T/C | SNP |  |  |  | *yafM/fhiA* (36/188) |  | • | • |
|  | 1259939 | T/C | SNP | *ychM* | 88 | T30A |  |  | • |  |
|  | 1492848 | T/C | SNP | *ydcI* | 248 | D83G |  |  | • | • |
|  | 1630183 | T/C | SNP | *ydfJ* | 127 | I43V |  |  | • | • |
|  | 1749334 | T/C | SNP | *ydhW* | 415 | T139A |  |  | • |  |
|  | 1786127 | A/G | SNP | *ydiA* | 659 | E220G |  |  | • | • |
|  | 1766716 | G/A | SNP |  |  |  | *ydiJ/ydiK (7/382)* |  | • | • |
|  | 1869851 | -/G | INS | *yeaI* | 1443 |  |  |  | • | • |
|  | 1882983 | G/A | SNP | *yeaW* | 295 | G99S |  |  | • | • |
|  | 2027827 | G/A | SNP | *yedA* | 265 | A89T |  |  |  | • |
|  | 2203311 | G/A | SNP | *yehL* | 694 | A232T |  |  |  | • |
|  | 2515043 | G/A | SNP | *yfeA* | 812 | T271M |  |  | • | • |
|  | 2628977 | A/G | SNP |  |  |  | *yfgI/guaA* (90/3) |  |  | • |
|  | 2646876 | G/A | SNP | *yfhM* | 3434 | A1145V |  |  | • |  |
|  | 2767665 | -/C | INS |  |  |  | *yfjQ/yfjR* (157/60) |  | • | • |
|  | 2993349 | G/A | SNP | *ygeM* | 419 | P140L |  |  |  | • |
|  | 3064285 | G/A | SNP | *ygfH* | 1462 | G488S |  |  | • | • |
|  | 3351624 | T/C | SNP | *yhcC* | 449 | H150R |  |  |  | • |
|  | 3637895 | T/- | DEL |  |  |  | *yhiO/uspA* (152/239) |  | • | • |
|  | 4198220 | T/C | SNP |  |  |  | *yjaG/hupA* (103/84) |  | • | • |
|  | 868537 | G/A | SNP | *yliA* | 1762 | A588T |  |  | • | • |
|  | 871599 | G/A | SNP | *yliD* | 487 | G163S |  |  | • | • |
|  | 1899328 | -/C | INS | *yoaE* | 282 |  |  |  | • | • |
|  | 2778387 | C/T | SNP | *ypjA* | 2362 | D788N |  |  | • | • |
|  | 2988177 | -/T | INS | *yqeK* | 206 |  |  |  | • | • |
|  |  |  |  |  |  |  |  | x2(*rrfE-insL*) | • | • |
|  |  |  |  |  |  |  |  | DEL(*insH-2 - insL-2*) | • | • |
|  |  |  |  |  |  |  |  | DEL(*glpF-rraA*) | • | • |

**Supplementary Table S3:** Oligonucleotide primers used in this study.

| **name** | **sequence** | **use** |
| --- | --- | --- |
| serA_FBR_A_F | CTTTAAAGTTAAGAGGCAAGAATGCATCATCACCATCACCACGCAAAGGTATCGCTGGAGAAAGACAAGATTAAG | Cloning of feedback resistant *serA* |
| serA_FBR_B | GAAGGACGTTGTCAAATTCACACAGCGG | Cloning of feedback resistant *serA* |
| serA_FBR_C | CCGCTGTGTGAATTTGACAACGTCCTTC | Cloning of feedback resistant *serA* |
| serA_FBR_D | GCGCGGCGATCGCGACGCCCTGCTCGGCGAAGATTTTGTTCAGCGCAGTTAGCACGCCCGGACGCGCTTCCGCGATGTGCATC | Cloning of feedback resistant *serA* |
| serA_FBR_E | GATGCACATCGCGGAAGCGCGTCCGGGCGTGCTAACTGCGCTGAACAAAATCTTCGCCGAGCAGGGCGTCGCGATCGCCGCGC | Cloning of feedback resistant *serA* |
| serA_FBR_F_R | CTCTTACGTGCCCGATCAACGCTAGCTTAGTACAGCAGACGGGCGCGAATGGTACCCGGAATAGCTTTCATTGCTTGCAGCGCTTTTTC | Cloning of feedback resistant *serA* |
| serB_A_F | CTTTAAAGTTAAGAGGCAAGAATGCATCATCACCATCACCACCCTAACATTACCTGGTGCGACCTGCC | Cloning of *serB* |
| serB_B_R | CTCTTACGTGCCCGATCAACGCTAGCTTACTTCTGATTCAGGCTGCCTGAGAGGATG | Cloning of *serB* |
| serC_A_F | CTTTAAAGTTAAGAGGCAAGAATGCATCATCACCATCACCACGCTCAAATCTTCAATTTTAGTTCTGGTCCGGCAATG | Cloning of *serC* |
| serC_B | GGTTTCATTCGGGCAATAGTGCATATAAGCAGCATTATC | Cloning of *serC* |
| serC_C | GATAATGCTGCTTATATGCACTATTGCCCGAATGAAACC | Cloning of *serC* |
| serC_D_R | CTCTTACGTGCCCGATCAACGCTAGCTTAACCGTGACGGCGTTCGAACTCAACC | Cloning of *serC* |
| sdaA_F | ATGCATCATCACCATCACCACATTAGTCTATTCGACATGTTTAAGGTGG | Cloning of *sdaA* |
| sdaA_R | CTCTTACGTGCCCGATCAACGCTAGCTTAGTCACACTGGACTTTGATTGCC | Cloning of *sdaA* |
| mgsA_A_F | CTTTAAAGTTAAGAGGCAAGAATGCATCATCACCATCACCACGAACTGACGACTCGCACTTTACCTGCG | Cloning of *mgsA* |
| mgsA_B_R | CTCTTACGTGCCCGATCAACGCTAGCTTACTTCAGACGGTCCGCGAGATAACGC | Cloning of *mgsA* |
| serA-pet-F | GGCCATATCGAAGGTCGTCATATGGCAAAGGTATCGCTGGAGAAAGACAAGATTAAG | Cloning of *serA* variants into pet16B |
| serA-pet-R | CTTTGTTAGCAGCCGGATCCTCGAGTTAGTACAGCAGACGGGCGCGAATGG | Cloning of *serA* variants into pet16B |
| serA_KO_F | CCTGCCCGTTGATTTTCAGAGAAGGGGAATTAGTACAGCAGACGGGCGCGAATTAACCCTCACTAAAGGGCG | *serA* KO-cassette amp. Gene Bridges, Km |
| serA_KO_R | GCGGATGCAAATCCGCACACAACATTTCAAAAGACAGGATTGGGTAAATGTAATACGACTCACTATAGGGCTC | *serA* KO-cassette amp. Gene Bridges, Km |
| sdaA_KO_F | TATTAGTTCGTTACTGGAAGTCCAGTCACCTTGTCAGGAGTATTATCGTGAATTAACCCTCACTAAAGGGCG | *sdaA* KO-cassette amp. Gene Bridges, Km |
| sdaA_KO_R | ATCCGTTGCAGATGGGCGAGTAAGAAGTATTAGTCACACTGGACTTTGATTAATACGACTCACTATAGGGCTC | *sdaA* KO-cassette amp. Gene Bridges, Km |
| sdaB_KO_F | GCCGCTTTCGGGCGGCGCTTCCTCCGTTTTAACGCGATGTATTTCCTATGAATTAACCCTCACTAAAGGGCG | *sdaB* KO-cassette amp. Gene Bridges, Km |
| sdaB_KO_R | CCTCGCAAAACGAGGCCTTTGGAGAGCGATTAATCGCAGGCAACGATCTTTAATACGACTCACTATAGGGCTC | *sdaB* KO-cassette amp. Gene Bridges, Km |
| tdcB_KO_F | GTACTCATATCCTATCCTCAACGAATTAATTAAGCGTCAACGAAACCGGTAATTAACCCTCACTAAAGGGCG | *tdcB* KO-cassette amp. Gene Bridges, Km |
| tdcB_KO_R | TCGGTTACGGTTACCTACATATTTAATTCAGGCGAAGAGGTTTTATAATGTAATACGACTCACTATAGGGCTC | *tdcB* KO-cassette amp. Gene Bridges, Km |
| tdcG_KO_F | GCACCCAAGGATGAAAGCTGACAGCAATGTCAGCCGCAGACCACTTTAATAATTAACCCTCACTAAAGGGCG | *tdcG* KO-cassette amp. Gene Bridges, Km |
| tdcG_KO_R | AGGTCGTTCCGCTCCACTTCACTGAACGGCAATCCGAGGGTGTGGATATGTAATACGACTCACTATAGGGCTC | *tdcG* KO-cassette amp. Gene Bridges, Km |
| mgsA_KO_K_F | AACAGGTGGCGTTTGCCACCTGTGCAATATTACTTCAGACGGTCCGCGAGAATTAACCCTCACTAAAGGGCG | *mgsA* KO-cassette amp. Gene Bridges, Km |
| mgsA_KO_K_R | TAAGTGCTTACAGTAATCTGTAGGAAAGTTAACTACGGATGTACATTATGTAATACGACTCACTATAGGGCTC | *mgsA* KO-cassette amp. Gene Bridges, Km |
| dld_KO_F | CGCTATTCTAGTTTGTGATATTTTTTCGCCACCACAAGGAGTGGAAAATGGTGTAGGCTGGAGCTGCTTC | *dld* KO-cassette amp. pKD3/4, CAP/Km |
| dld_KO_R | GGATGGCGATACTCTGCCATCCGTAATTTTTACTCCACTTCCTGCCAGTTCATATGAATATCCTCCTTAG | *dld* KO-cassette amp. pKD3/4, CAP/Km |
| gldA_KO_F | CGACTGGAATGCCGCATTTGGCACTACTCATCTCTAAAGGAGCAATTATGGTGTAGGCTGGAGCTGCTTC | *gldA* KO-cassette amp. pKD3/4, CAP/Km |
| gldA_KO_R | TCCCGGACAAGCCGGGAGTTTGGAGTAGGTTATTCCCACTCTTGCAGGAACATATGAATATCCTCCTTAG | *gldA* KO-cassette amp. pKD3/4, CAP/Km |
| gloA_KO_F | TACTAAAACAACATTTTGAATCTGTTAGCCATTTTGAGGATAAAAAGATGGTGTAGGCTGGAGCTGCTTC | *gloA* KO-cassette amp. pKD3/4, CAP/Km |
| gloA_KO_R | GGCGCGATGAGTTCACGCCCGGCAGGAGATTAGTTGCCCAGACCGCGACCCATATGAATATCCTCCTTAG | *gloA* KO-cassette amp. pKD3/4, CAP/Km |
| aldA_KO_F | AACAATGTATTCACCGAAAACAAACATATAAATCACAGGAGTCGCCCATGGTGTAGGCTGGAGCTGCTTC | *aldA* KO-cassette amp. pKD3/4, CAP/Km |
| aldA_KO_R | GAGGAAAAAACCTCCGCCTCTTTCACTCATTAAGACTGTAAATAAACCACCATATGAATATCCTCCTTAG | *aldA* KO-cassette amp. pKD3/4, CAP/Km |
| hchA_KO_C_F | CGCAAATATAGTGACTACCCTAACTAAGCAACAATAAGGAATACACTATGGTGTAGGCTGGAGCTGCTTC | *hchA* KO-cassette amp. pKD3/4, CAP/Km |
| hchA_KO_C_R | TATGCGCTTACATTCAAACGTAACAGGGATTAACCCGCGTAAGCTGCCAGCATATGAATATCCTCCTTAG | *hchA* KO-cassette amp. pKD3/4, CAP/Km |
| ppsA_KO_C_F | CGGCGACTAAACGCCGCCGGGGATTTATTTTATTTCTTCAGTTCAGCCAGGTGTAGGCTGGAGCTGCTTC | *ppsA* KO-cassette amp. pKD3/4, CAP/Km |
| ppsA_KO_C_R | AGAAATGTGTTTCTCAAACCGTTCATTTATCACAAAAGGATTGTTCGATGCATATGAATATCCTCCTTAG | *ppsA* KO-cassette amp. pKD3/4, CAP/Km |
| serA_KO_Ver_F | CTCAACATCGCGACGCAAAC | PCR verification of *serA* deletion |
| serA_KO_Ver_R | TCTGGAGCAGACTCGCAAAG | PCR verification of *serA* deletion |
| tdcG_KO_Ver_F | CTTATTATTTTTTTCCGAGCCGCATCAAGGCGATATG | PCR verification of *tdcG* deletion |
| tdcG_KO_Ver_R | CGTGTTTATCACCGATCTGAATGATTTTGCCAC | PCR verification of *tdcG* deletion |
| tdcB_KO_Ver_F | CGATTGCCGTACCAAACAAGCC | PCR verification of *tdcB* deletion |
| tdcB_KO_Ver_R | AGCAGCATCGGTTTTGGTGGAA | PCR verification of *tdcB* deletion |
| sdaB_KO_Ver_F | GGGTCTGATTGCAATCTCCGCA | PCR verification of *sdaB* deletion |
| sdaB_KO_Ver_R | ATGAACAGCCACGATAACCCCC | PCR verification of *sdaB* deletion |
| sdaA_KO_Ver_F | GGCGCTGCAAATTGGTGTGAAA | PCR verification of *sdaA* deletion |
| sdaA_KO_Ver_R | CCTGACGCAACAGTGGAAGTGT | PCR verification of *sdaA* deletion |
| mgsA_KO_Ver_F | CACCGCAGTCTCAGGTGCTCAC | PCR verification of *mgsA* deletion |
| mgsA_KO_Ver_R | CTGACCCGGGCACGCCATCG | PCR verification of *mgsA* deletion |
| dld_KO_Ver_F | TTCTTCCTTTGTTGCCCGACGT | PCR verification of *dld* deletion |
| dld_KO_Ver_R | TAGTGATGGACGCGTTTGGCAA | PCR verification of *dld* deletion |
| gldA_KO_Ver_F | CGGCCTACAAAAGCACGCAAAT | PCR verification of *gldA* deletion |
| gldA_KO__Ver_R | CACCCTGCCCTTAGATGTAGCG | PCR verification of *gldA* deletion |
| gloA_KO_Ver_F | GTAATCCAACATTGCGAGCGGC | PCR verification of *gloA* deletion |
| gloA_KO_Ver_R | TCCATTTTCAGGGTGATGGCGG | PCR verification of *gloA* deletion |
| aldA_KO_Ver_F | CCACTTGTTTGCAAACGGGCAT | PCR verification of *aldA* deletion |
| aldA_KO_Ver_R | GTTTGATGCCACGCAAACGGAA | PCR verification of *aldA* deletion |
| hchA_KO_Ver_F | CAGCACTAAATCTCTCCCCGCC | PCR verification of *hchA* deletion |
| hchA_KO_Ver_R | CGTAGGTCAGGGACTAGGCCTT | PCR verification of *hchA* deletion |
| ppsA_KO_Ver_F | TCTCTGCCGTATGGATGAGGCT | PCR verification of *ppsA* deletion |
| ppsA_KO_Ver_R | GCGTGTCCTAATACCTCCGCAG | PCR verification of *ppsA* deletion |
| SdaA-ProEx-F | CCGCAGGCATAATTCGTGAGCTGGCGCTGCAAATTGGTGTGAAACCCTGAAATTAACCCTCACTAAAGGGCGGAGCTGCTTCGAAGTTC | *sdaA* promoter exchange |
| CAP sdaA-R | TAATACGACTCACTATAGGGCTCCATATGAATATCCTCCTTAG | *sdaA* promoter exchange |
| pS-bridge | GAGCCCTATAGTGAGTCGTATTAAATACTTGACATATCACTGTGATTCACATATAATATGCG | *sdaA* / serA promotor exchange |
| SdaA-ProEx-R | GAAGATGAGGGACCAATCCCCACCTTAAACATGTCGAATAGACTAATCATTCTTGCCTCTTAACTTTAAAGTTAAACAAAATTATTTCTATTAACTAGTGAATTC | *sdaA* promoter exchange |
| serA*-ProEx-F | AATTAACCCTCACTAAAGGGCGGAGCTGC | *serA* promoter exchange |
| CAP-SerA*-R | CTAAGGAGGATATTCATATGGAGCCCTATAGTGAGTCGTATTA | *serA* promoter exchange |
| serA*-ProEx-R | TTAGTACAGCAGACGGGCGCGAATGG | *serA* promoter exchange |
| 2660_sdaA_Pro_V_F | GCCAGTGAAGATGAAGTCTC | PCR verification of *sdaA* promotor exchange |
| 2661_sdaA_Pro_V_R | ACAGTGAACCATAAACGTCC | PCR verification of *sdaA* promotor exchange |
| mgsA_qPCR_F | GCACACGATCACTGCAAAC | 190 bp amplicon for *mgsA* qPCR |
| mgsA_qPCR_R | CTTCTGAGATCAATGCGCC | 190 bp amplicon for *mgsA* qPCR |
| serA_qPCR_F | AAGAATCCATCCGCGATGCC | 107 bp amplicon for *serA* qPCR |
| serA_qPCR_R | ACAGAAACAGCCAATAGCGAC | 107 bp amplicon for *serA* qPCR |
| serB_qPCR_F | GACCCAATACCAGAGCAAAC | 197 bp amplicon for *serB* qPCR |
| serB_qPCR_R | ATCCATCACCAGCAAACCC | 197 bp amplicon for *serB* qPCR |
| serC_qPCR_F | TTAAACAGGCTCAACAGGAAC | 290 bp amplicon for *serC* qPCR |
| serC_qPCR_R | CGTGCAGTATTTTTTCGCTTC | 290 bp amplicon for *serC* qPCR |
| sdaA_qPCR_F | GTCCCTCATCTTCCCATACC | 283 bp amplicon for *sdaA* qPCR |
| sdaA_qPCR_R | AAATCCACTTCATGCCGTC | 283 bp amplicon for *sdaA* qPCR |
| rrsA_qPCR_F | CTCTTGCCATCGGATGTGCCCA | Reference gene for microbial qPCR |
| rrsA_qPCR_R | CCAGTGTGGCTGGTCATCCTCTCA | Reference gene for microbial qPCR |

**Supplementary Method to the Computational analysis to identify glycolytic bypasses in *E. coli* / A constraint-based method for finding glycolysis bypasses**

**Alterations to the iML1515 model**

We made a few changes to the genome-scale model of *E. coli* (iML1515) (*1*):

- Removing all non-cytoplasmic reactions (i.e. exchange or transport reactions), except for exchange reactions of inorganic metabolites: protons, water, orthophosphate, ammonium, and oxygen
- Removing all boundary reactions (i.e. sink reactions needed to allow certain co-factors to leave the system)
- Replacing all flavoredoxins and thioredoxins with NADP(H): We replaced all flavoredoxins and thioredoxins with NADP(H), since we do not have a good estimate of their reduction potential, and therefore the MDF for pathways using them was artificially high. We can assume that the electrons used for reducing CO2 in the carbon fixation cycle ultimately have to pass through NADPH, and therefore a simple solution was to replace the electron donor with NADPH. This way, we could keep the flavoredoxins/thioredoxins-dependent reactions in the model while having a more realistic estimate of their thermodynamics.
- Removing the formate-tetrahydrofolate ligase reaction (FTHFLi): We found that the reaction formate-tetrahydrofolate ligase (FTHFLi) appears in some of the solutions although the gene associated with this reaction is unknown. FTHFLi was thus excluded from our model altogether. Notably, removing this reaction does not significantly affect the space of solutions, because it can be easily replaced by GAR transformylase-T (GART) and the reverse reaction of Phosphoribosylglycinamide formyltransferase (GARFT).
- Add the objective reaction (OBJ): glycerol + n ADP + n Pi → pyruvate + n ATP + n H2O
  where "n" refers to the ATP yield. In this study, the allowed values were -1, 0, and 1.
- Setting the bounds (the range of possible fluxes) of all remaining reactions to be between -10 and 10

**Supplementary Table S4. The allowed concentration ranges for metabolites in the model.**

| **Compound** | **BiGG identifier** | **Concentration range** |
| --- | --- | --- |
| ATP | atp_c | 5 mM |
| ADP | adp_c | 0.5 mM - 2.5 mM |
| AMP | amp_c | 0.5 mM - 2.5 mM |
| NAD+ | nad_c | 1 mM |
| NADH | nadh_c | 10 μM - 100 μM |
| NADP+ | nadp_c | 10 μM |
| NADPH | nadph_c | 10 μM - 100 μM |
| O2 | o2_c | 273 μM |
| CO2 | co2_c | 6.3 mM |
| CoA | coa_c | 1 mM - 5 mM |
| orthophosphate | pi_c | 1 mM - 10 mM |
| pyrophosphate | ppi_c | 0.5 mM - 1.5 mM |
| ammonia | nh4_c | 1 mM - 10 mM |
| alpha-ketoglutarate | akg_c | 0.5 mM - 5 mM |
| glutamate | glu__L_c | 30 mM - 150 mM |

Note: All metabolites that do not appear in this table were constrained by the default ranges of 1 μM to 10 mM.

**Supplementary Table S5:** RNA samples & Reverse transcription information. (A) Sample information section: Preculture, carbon source on which the strains have been cultured in minimal medium (M9); Rep., biological replicate number; Strain, sampled strain; ODharvest, the optical density of the culture when harvested; RNA(ng/µL), nucleic acid concentration; A260/280, purity. The RT mix section describes the components and aliquots used for reverse transcription of each sample: a template volume equivalent to 500ng of RNA has been used in each reaction. RT- mix section: the same amounts of the template have been used for the RT- negative control applying the same dilution factor. (B) Reverse transcription PCR program according to kit manufacturer instructions.

**A**

| **Sample information** | | | | | | **RT mix** | | | | | | **RT- mix** | | |
| --- | --- | --- | --- | --- | --- | --- | --- | --- | --- | --- | --- | --- | --- | --- |
| **Preculture** | **Rep.** | **Strain** | **OD_harvest_** | **RNA (ng/μL)** | **A_260_/A_280_** | **RNA (500ng)** | **H_2_O μL** | **RT Buffer** | **μL RT enzyme** | **Volume (μL)** | **Dilution Factor** | **RNA (500ng)** | **H_2_O μL** | **Final volume** |
| Glycerol 20mM | 1 | SIJ488 | 0.67 | 271.00 | 2.12 | 1.85 | 13.15 | 4 | 1 | 20 | 10.84 | 1.85 | 18.15 | 20.00 |
|  | 2 | SIJ488 | 0.63 | 234.10 | 2.10 | 2.14 | 12.86 | 4 | 1 | 20 | 9.36 | 2.14 | 17.86 | 20.00 |
|  | 3 | SIJ488 | 0.68 | 429.20 | 2.08 | 1.16 | 13.84 | 4 | 1 | 20 | 17.17 | 1.16 | 18.84 | 20.00 |
|  | 1 | ΔtpiA | 0.6 | 228.50 | 2.12 | 2.19 | 12.81 | 4 | 1 | 20 | 9.14 | 2.19 | 17.81 | 20.00 |
|  | 2 | ΔtpiA | 0.56 | 117.00 | 2.03 | 4.27 | 10.73 | 4 | 1 | 20 | 4.68 | 4.27 | 15.73 | 20.00 |
|  | 3 | ΔtpiA | 0.64 | 136.00 | 2.11 | 3.68 | 11.32 | 4 | 1 | 20 | 5.44 | 3.68 | 16.32 | 20.00 |
| Glycerol 4mM Succinate 40mM | 1 | SIJ488 | 0.52 | 156.20 | 1.96 | 3.20 | 11.80 | 4 | 1 | 20 | 6.25 | 3.20 | 16.80 | 20.00 |
|  | 2 | SIJ488 | 0.56 | 195.80 | 2.00 | 2.55 | 12.45 | 4 | 1 | 20 | 7.83 | 2.55 | 17.45 | 20.00 |
|  | 3 | SIJ488 | 0.52 | 202.50 | 1.92 | 2.47 | 12.53 | 4 | 1 | 20 | 8.10 | 2.47 | 17.53 | 20.00 |
|  | 1 | ΔtpiA | 0.46 | 231.9 | 1.93 | 2.16 | 12.84 | 4 | 1 | 20 | 9.28 | 2.16 | 17.84 | 20.00 |
|  | 2 | ΔtpiA | 0.62 | 366.4 | 2.05 | 1.36 | 13.64 | 4 | 1 | 20 | 14.66 | 1.36 | 18.64 | 20.00 |
|  | 3 | ΔtpiA | 0.6 | 289.2 | 2.08 | 1.73 | 13.27 | 4 | 1 | 20 | 11.57 | 1.73 | 18.27 | 20.00 |
|  | 1 | Δpgk | 0.52 | 141.4 | 2.09 | 3.54 | 11.46 | 4 | 1 | 20 | 5.66 | 3.54 | 16.46 | 20.00 |
|  | 2 | Δpgk | 0.55 | 181.9 | 1.84 | 2.75 | 12.25 | 4 | 1 | 20 | 7.28 | 2.75 | 17.25 | 20.00 |
|  | 3 | Δpgk | 0.48 | 183.5 | 1.91 | 2.72 | 12.28 | 4 | 1 | 20 | 7.34 | 2.72 | 17.28 | 20.00 |
| Glycerol 20mM | 1 | SIJ488 | 0.73 | 412.90 | 2.12 | 1.21 | 13.79 | 4 | 1 | 20 | 16.52 | 1.21 | 18.79 | 20.00 |
|  | 2 | SIJ488 | 0.61 | 223.40 | 2.06 | 2.24 | 12.76 | 4 | 1 | 20 | 8.94 | 2.24 | 17.76 | 20.00 |
|  | 3 | SIJ488 | 0.74 | 425.20 | 2.02 | 1.18 | 13.82 | 4 | 1 | 20 | 17.01 | 1.18 | 18.82 | 20.00 |
|  | 1 | iso1 | 0.62 | 210.00 | 2.10 | 2.38 | 12.62 | 4 | 1 | 20 | 8.40 | 2.38 | 17.62 | 20.00 |
|  | 2 | iso1 | 0.79 | 337.00 | 2.08 | 1.48 | 13.52 | 4 | 1 | 20 | 13.48 | 1.48 | 18.52 | 20.00 |
|  | 3 | iso1 | 0.95 | 275.10 | 2.07 | 1.82 | 13.18 | 4 | 1 | 20 | 11.00 | 1.82 | 18.18 | 20.00 |

**B**

| **RT program** | | |
| --- | --- | --- |
| **Step** | **Temp (°C)** | **Time (mins)** |
| Annealing | 22 | 5 |
| Elongation | 42 | 30 |
| Inactivation | 85 | 5 |

**Supplementary References**

1. J. M. Monk, C. J. Lloyd, E. Brunk, N. Mih, A. Sastry, Z. King, R. Takeuchi, W. Nomura, Z. Zhang, H. Mori, A. M. Feist, B. O. Palsson, iML1515, a knowledgebase that computes *Escherichia coli* traits. *Nat Biotechnol* **35**, 904-908 (2017).
