# Supplementary material for: Activating Silent Glycolysis Bypasses in Escherichia coli
Source: Biodes Res. 2022 May 11;2022:9859643. doi: 10.34133/2022/9859643 (PMC10521649; doi:10.34133/2022/9859643)

Primers	Amp. bp	Target	Coordinates
mgsA	190	mgsA	1,026,775 - 1,026,964
serA	107	serA	3,058,162 - 3,058,268
serB	193	serB	4,625,050 - 4,625,242
serC	290	serC	957,708 - 957,997
sdaA	283	sdaA	1,896,969 - 1,897,251
16S rRNA	105	rrsA	4,035,745 - 4,035,849
	105	rrsB	4,166,873 - 4,166,977
	105	rrsD	3,428,444 - 3,428,548
	105	rrsE	4,208,361 - 4,208,465
	105	rrsG	2,730,839 - 2,730,943
	105	rrsH	223,985 - 224,089

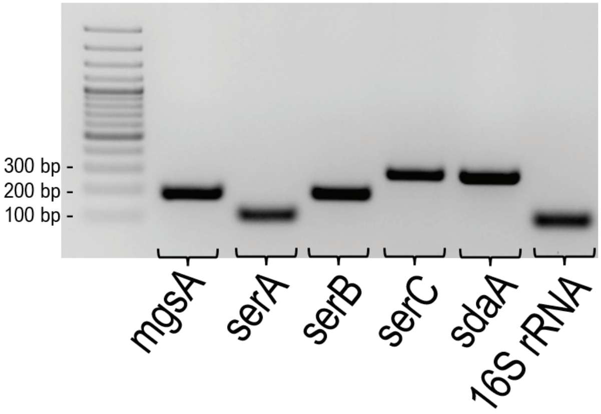

Supplement: Supplementary Materials — Supplementary Figures in GitLab: computationally identified EMP bypasses. Figure S1: growth of a Δtpi ΔmgsA strain on glycerol and succinate compared to a Δtpi strain. Figure S2: mgsA transcript levels determined by qPCR experiments. Figure S3: predicted and measured 13C-labeling in selected amino acids upon feeding of 1,6-13C2-glucose in cells using EMP-glycolysis, the methylglyoxal pathway, or the serine shunt. Figure S4: genome sequencing coverage of serine-tolerant Δeno isolates (G3 mutants). Figure S5: transcript level of serine shunt genes of the glycerol evolved iso1 strain. Figure S6: target specificity analysis of qPCR primers. Figure S7: serine-dependent growth of iso1 ΔserA strain compared to a WT-based ΔserA strain. Table S1: identified mutations different in the serine-tolerant Δeno strains compared to the reference strain. Table S2: identified mutations in the evolved Δ eno strains. Table S3: oligonucleotide primers used. Supplementary Method to the computational analysis to identify glycolytic bypasses in E. coli/A constraint-based method for finding glycolysis bypasses. Table S4. Allowed metabolite concentration ranges in the model. Table S5: RNA samples and reverse transcription information. [file 9859643.f1.zip › S6.pdf]
