# Supplementary material for: Activating Silent Glycolysis Bypasses in Escherichia coli
Source: Biodes Res. 2022 May 11;2022:9859643. doi: 10.34133/2022/9859643 (PMC10521649; doi:10.34133/2022/9859643)
Supplement: Supplementary Materials — Supplementary Figures in GitLab: computationally identified EMP bypasses. Figure S1: growth of a Δtpi ΔmgsA strain on glycerol and succinate compared to a Δtpi strain. Figure S2: mgsA transcript levels determined by qPCR experiments. Figure S3: predicted and measured 13C-labeling in selected amino acids upon feeding of 1,6-13C2-glucose in cells using EMP-glycolysis, the methylglyoxal pathway, or the serine shunt. Figure S4: genome sequencing coverage of serine-tolerant Δeno isolates (G3 mutants). Figure S5: transcript level of serine shunt genes of the glycerol evolved iso1 strain. Figure S6: target specificity analysis of qPCR primers. Figure S7: serine-dependent growth of iso1 ΔserA strain compared to a WT-based ΔserA strain. Table S1: identified mutations different in the serine-tolerant Δeno strains compared to the reference strain. Table S2: identified mutations in the evolved Δ eno strains. Table S3: oligonucleotide primers used. Supplementary Method to the computational analysis to identify glycolytic bypasses in E. coli/A constraint-based method for finding glycolysis bypasses. Table S4. Allowed metabolite concentration ranges in the model. Table S5: RNA samples and reverse transcription information. [file 9859643.f1.zip › Supplementary Material qPCRs Analysis + RT-controls.pdf]

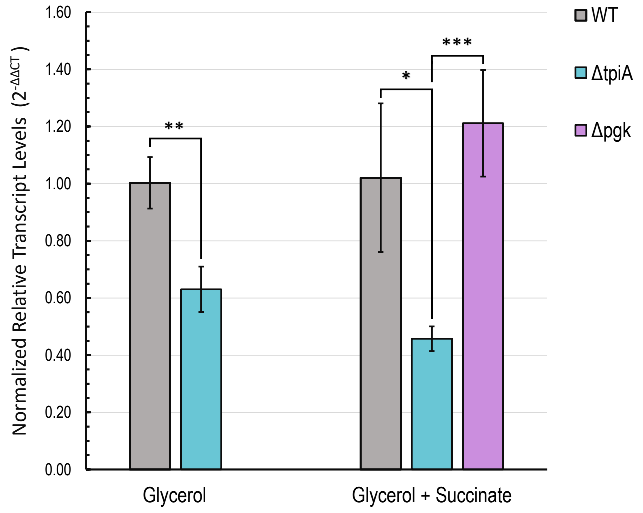

Product from sample x	Avg. tech. rep.
mgsA-WT-1	27.3252
mgsA-WT-2	28.1001
mgsA-WT-3	28.3822
mgsA- ΔtpiA	28.1571
mgsA- ΔtpiA	29.3771
mgsA- ΔtpiA	29.8755
16S-WT-1	12.0051
16S-WT-2	12.5460
16S-WT-3	13.0557
16S- ΔtpiA	12.1452
16S- ΔtpiA	13.0917
16S- ΔtpiA	13.9513

mgsA (Glycerol)	ΔCq	ΔΔCq	2^{-DDCq}
WT-1	15.3201	-0.0801	1.1
WT-2	15.5540	0.1538	0.9
WT-3	15.3266	-0.0737	1.1
ΔtpiA-1	16.0119	0.6117	0.7
ΔtpiA-2	16.2854	0.8852	0.5
ΔtpiA-3	15.9242	0.5239	0.7
**WT Avg.**	**15.4002**		

mgsA (Glycerol)		
**Sample**	**2^-ΔΔCq Avg.**	**St.Dev**
WT	1.0027936	0.09
ΔtpiA	0.630443	0.08

Ttest	P-Value
WT vs ΔtpiA	0.0058386

Differential expression level of DtpiA & WT on Glycerol (above) and Negative controls (belo

RT- samples		gDNA contamination signal		
**Product from RT- sample x**	**Avg. tech. rep.**	**ΔCt**	**ΔCt fold diff.**	**gDNA signal %**
mgsA-WT-1	36.1282	8.8030	446.6	0.2
mgsA-WT-2	32.1124	4.0123	16.1	6.2
mgsA-WT-3	32.1373	3.7551	13.5	7.4
mgsA-ΔtpiA-1	31.9332	3.7760	13.7	7.3
mgsA-ΔtpiA-2	30.4723	1.0952	2.1	46.8
mgsA-ΔtpiA-3	32.5090	2.6335	6.2	0.0
16S-WT-1	28.6104	16.6053	99699.7	0.0
16S-WT-2	29.1174	16.5714	97383.7	0.0
16S-WT-3	28.4905	15.4348	44294.5	0.0
16S-ΔtpiA-1	29.0798	16.9346	125260.4	0.0
16S-ΔtpiA-2	27.7673	14.6757	26170.7	0.0
16S-ΔtpiA-3	28.5670	14.6157	25104.8	0.0

NTC controls	
**mgsA-H_2O**	#DIV/0!
**16S-H2O**	32.8716

Product from sample x	Avg. tech. rep.
mgsA-WT-1	25.8786
mgsA-WT-2	26.0372
mgsA-WT-3	25.2663
mgsA-ΔtpiA-1	26.0648
mgsA-ΔtpiA-2	25.7631
mgsA-ΔtpiA-3	26.6107
mgsA-Δpgk-1	24.9996
mgsA-Δpgk-2	25.2100
mgsA-Δpgk-3	25.6801
16S-WT-1	10.7016
16S-WT-2	10.7311
16S-WT-3	10.6213
16S-ΔtpiA-1	10.0392
16S-ΔtpiA-2	9.5457
16S-ΔtpiA-3	10.3275
16S-Δpgk-1	10.4607
16S-Δpgk-2	10.3831
16S-Δpgk-3	10.7147

mgsA (G+S)	ΔCq	ΔΔCq	2^{-DDCq}
WT-1	15.1769	0.1342	0.9
WT-2	15.3062	0.2635	0.8
WT-3	14.6450	-0.3977	1.3
ΔtpiA-1	16.0256	0.9829	0.5
ΔtpiA-2	16.2174	1.1747	0.4
ΔtpiA-3	16.2832	1.2405	0.4
Δpgk-1	14.5389	-0.5038	1.4
Δpgk-2	14.8269	-0.2158	1.2
Δpgk-3	14.9655	-0.0772	1.1
**WT Avg.**	**15.0427**		

mgsA (G+S)		
Sample	2^{-DDCq} Avg.	St.Dev
WT	0.87	0.51
ΔtpiA	0.76	0.49
Δpgk	1.00	0.52

Ttest	P-Value
WT vs ΔtpiA	0.02083
WT vs Δpgk	0.35995
ΔtpiA vs Δpgk	0.00242

Differential expression level of WT, Dpgk and DtpiA on Glycerol+Succinate (above) and Negative controls (below)

RT- controls		gDNA contamination signal		
Product from RT- sample x	Avg. tech. rep.	ΔCt	ΔCt fold diff.	gDNA signal %
mgsA1-WT-1	30.1240	4.2455	19.0	5.3
mgsA1-WT-2	30.5350	4.4977	22.6	4.4
mgsA1-WT-3	31.1281	5.8618	58.2	1.7
mgsA-ΔtpiA-1	29.6779	3.6131	12.2	8.2
mgsA-ΔtpiA-2	30.7484	4.9853	31.7	3.2
mgsA-ΔtpiA-3	30.8759	4.2652	19.2	5.2
mgsA-Δpgk-1	29.5617	4.5621	23.6	4.2
mgsA-Δpgk-2	30.7509	5.5409	46.6	2.1
mgsA-Δpgk-3	29.7196	4.0395	16.4	6.1
16S-WT-1	28.1901	17.4885	183892.5	0.0
16S-WT-2	28.3275	17.5965	198181.9	0.0
16S-WT-3	28.8568	18.2355	308631.9	0.0
16S-ΔtpiA-1	28.3611	18.3219	327679.7	0.0
16S-ΔtpiA-2	28.4207	18.8751	480793.1	0.0
16S-ΔtpiA-3	28.6946	18.3671	338104.7	0.0
16S-Δpgk-1	28.0530	17.5923	197608.6	0.0
16S-Δpgk-2	27.9447	17.5616	193450.6	0.0
16S-Δpgk-3	27.6715	16.9568	127206.5	0.0

NTC controls	
**mgsA-H_2O**	#DIV/0!
**16S-H2O**	32.8716

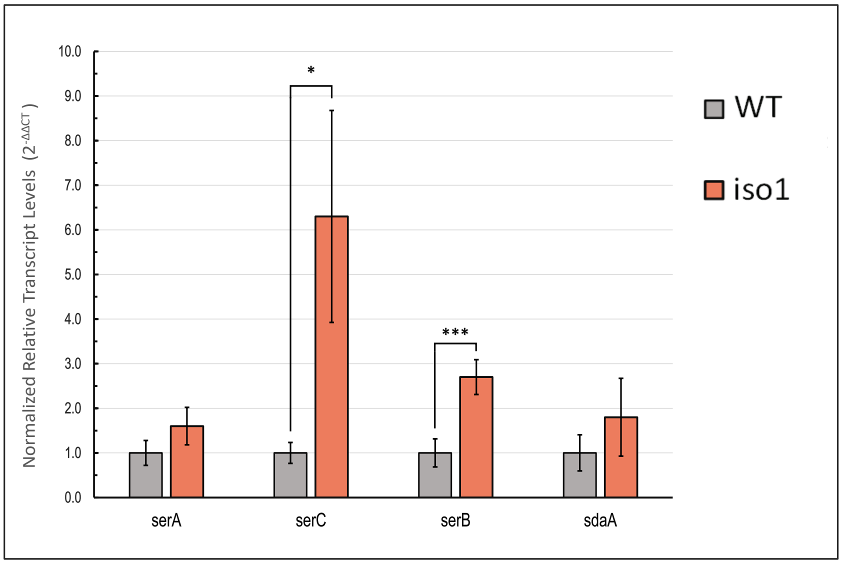

Differential Gene expression between WT and iso1

Product from sample x	Avg. tech. rep.
16S-SIJ488 1	14.2334
16S-SIJ488 2	14.9425
16S-SIJ488 3	14.5587
16S-iso1-1	14.1741
16S-iso1-2	16.0900
16S-iso1-3	13.5790
serA-SIJ488 1	28.1958
serA-SIJ488 2	28.1005
serA-SIJ488 3	27.8662
serA-iso1-1	27.3670
serA-iso1-2	28.8583
serA-iso1-3	26.0143
serC-SIJ488 1	29.9540
serC-SIJ488 2	30.0487
serC-SIJ488 3	29.6379
serC-iso1-1	27.0645
serC-iso1-2	29.1917
serC-iso1-3	25.7072
serB-SIJ488 1	31.1916
serB-SIJ488 2	31.0361
serB-SIJ488 3	30.6728
serB-iso1-1	29.3337
serB-iso1-2	31.1220
serB-iso1-3	28.3349
sdaA-SIJ488 1	30.7186
sdaA-SIJ488 2	30.2514
sdaA-SIJ488 3	30.0033
sdaA-iso1-1	30.1197
sdaA-iso1-2	30.8741
sdaA-iso1-3	27.9471

serA	ΔCq	ΔΔCq	2^{-DDCq}
SIJ488 1	13.96231	0.4864	0.7
SIJ488 2	13.15803	-0.3179	1.2
SIJ488 3	13.30743	-0.1685	1.1
iso1-1	13.19288	-0.2830	1.2
iso1-2	12.76826	-0.7077	1.6
iso1-3	12.43533	-1.0406	2.1
**WT AVERAGE**	**13.47592**		

serA		
Sample	2^{-DDCq} Avg.	St.Dev
WT	1.0	0.28
iso1	1.6	0.42

T.Test	P values
WT vs iso1	0.10523

serC	ΔCq	ΔΔCq	2^{-DDCq}
SIJ488 1	15.72052	0.4186	0.7
SIJ488 2	15.10626	-0.1957	1.1
SIJ488 3	15.07912	-0.2229	1.2
iso1-1	12.8904	-2.4116	5.3
iso1-2	13.10165	-2.2003	4.6
iso1-3	12.12822	-3.1737	9.0
**WT AVERAGE**	**15.30197**		

serC		
Sample	2^{-DDCq} Avg.	St.Dev
WT	1.0	0.24
iso1	6.3	2.38

T.Test	P values
WT vs iso1	0.01844

serB	ΔCq	ΔΔCq	2^{-DDCq}
SIJ488 1	16.95811	0.5695	0.7
SIJ488 2	16.09362	-0.2950	1.2
SIJ488 3	16.11409	-0.2745	1.2
iso1-1	15.15961	-1.2290	2.3
iso1-2	15.03196	-1.3566	2.6
iso1-3	14.75594	-1.6327	3.1
**WT AVERAGE**	**16.3886**		

serB		
Sample	2^{-DDCq} Avg.	St.Dev
WT	1.0	0.31
iso1	2.7	0.39

T.Test	P values
WT vs iso1	0.00485

sdaA	ΔCq	ΔΔCq	2^{-DDCq}
SIJ488 1	16.4852	0.7390	0.6
SIJ488 2	15.30893	-0.4373	1.4
SIJ488 3	15.44453	-0.3017	1.2
iso1-1	15.94567	0.1994	0.9
iso1-2	14.78412	-0.9621	1.9
iso1-3	14.36809	-1.3781	2.6
**WT AVERAGE**	**15.74622**		

sdaA		
Sample	2^{-DDCq} Avg.	St.Dev
WT	1.1	0.41
iso1	1.8	0.87

T.Test	P values
WT vs iso1	0.25153

Product from RT-sample x	Avg. tech. rep.	ΔCq	ΔCq fold diff.	gDNA signal %
16S-SIJ488 1	28.2346	14.0011	16396.6	0.0
16S-SIJ488 2	28.9439	14.0014	16399.9	0.0
16S-SIJ488 3	28.0478	13.4891	11497.7	0.0
16S-iso1-1	28.8570	14.6829	26303.0	0.0
16S-iso1-2	28.9768	12.8868	7573.7	0.0
16S-iso1-3	28.9593	15.3803	42651.4	0.0
serA-SIJ488 1	30.8060	2.6102	6.1	16.4
serA-SIJ488 2	32.2457	4.1452	17.7	5.7
serA-SIJ488 3	31.2426	3.3764	10.4	9.6
serA-iso1-1	33.6535	6.2866	78.1	1.3
serA-iso1-2	32.8597	4.0014	16.0	6.2
serA-iso1-3	32.8380	6.8237	113.3	0.9
serC-SIJ488 1	32.7542	2.8002	7.0	14.4
serC-SIJ488 2	34.4484	4.3997	21.1	4.7
serC-SIJ488 3	32.7843	3.1464	8.9	11.3
serC-iso1-1	35.5232	8.4588	351.8	0.3
serC-iso1-2	33.6636	4.4720	22.2	4.5
serC-iso1-3	33.6898	7.9826	252.9	0.4
serB-SIJ488 1	33.2026	2.0110	4.0	24.8
serB-SIJ488 2	33.3797	2.3436	5.1	19.7
serB-SIJ488 3	34.1062	3.4334	10.8	9.3
serB-iso1-1	35.7765	6.4428	87.0	1.1
serB-iso1-2	33.5530	2.4311	5.4	18.5
serB-iso1-3	33.6175	5.2826	38.9	2.6
sdaA-SIJ488 1	32.2984	1.5798	3.0	33.5
sdaA-SIJ488 2	32.5355	2.2841	4.9	20.5
sdaA-SIJ488 3	33.3772	3.3740	10.4	9.6
sdaA-iso1-1	33.1604	3.0407	8.2	12.2
sdaA-iso1-2	32.9161	2.0420	4.1	24.3
sdaA-iso1-3	32.9179	4.9708	31.4	3.2

NT controls

Primer	Cq
16S	29.1867
serA	34.02014
serC	35.05957
serB	37.7318
sdaA	33.08921
