# Supplementary figures and images for: Activating Silent Glycolysis Bypasses in Escherichia coli
Source: Biodes Res. 2022 May 11;2022:9859643. doi: 10.34133/2022/9859643 (PMC10521649; doi:10.34133/2022/9859643)

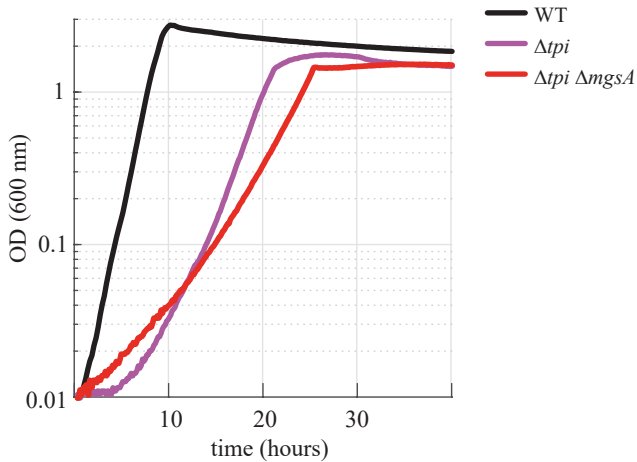

Supplement: Supplementary Materials — Supplementary Figures in GitLab: computationally identified EMP bypasses. Figure S1: growth of a Δtpi ΔmgsA strain on glycerol and succinate compared to a Δtpi strain. Figure S2: mgsA transcript levels determined by qPCR experiments. Figure S3: predicted and measured 13C-labeling in selected amino acids upon feeding of 1,6-13C2-glucose in cells using EMP-glycolysis, the methylglyoxal pathway, or the serine shunt. Figure S4: genome sequencing coverage of serine-tolerant Δeno isolates (G3 mutants). Figure S5: transcript level of serine shunt genes of the glycerol evolved iso1 strain. Figure S6: target specificity analysis of qPCR primers. Figure S7: serine-dependent growth of iso1 ΔserA strain compared to a WT-based ΔserA strain. Table S1: identified mutations different in the serine-tolerant Δeno strains compared to the reference strain. Table S2: identified mutations in the evolved Δ eno strains. Table S3: oligonucleotide primers used. Supplementary Method to the computational analysis to identify glycolytic bypasses in E. coli/A constraint-based method for finding glycolysis bypasses. Table S4. Allowed metabolite concentration ranges in the model. Table S5: RNA samples and reverse transcription information. [file 9859643.f1.zip › S1.pdf]

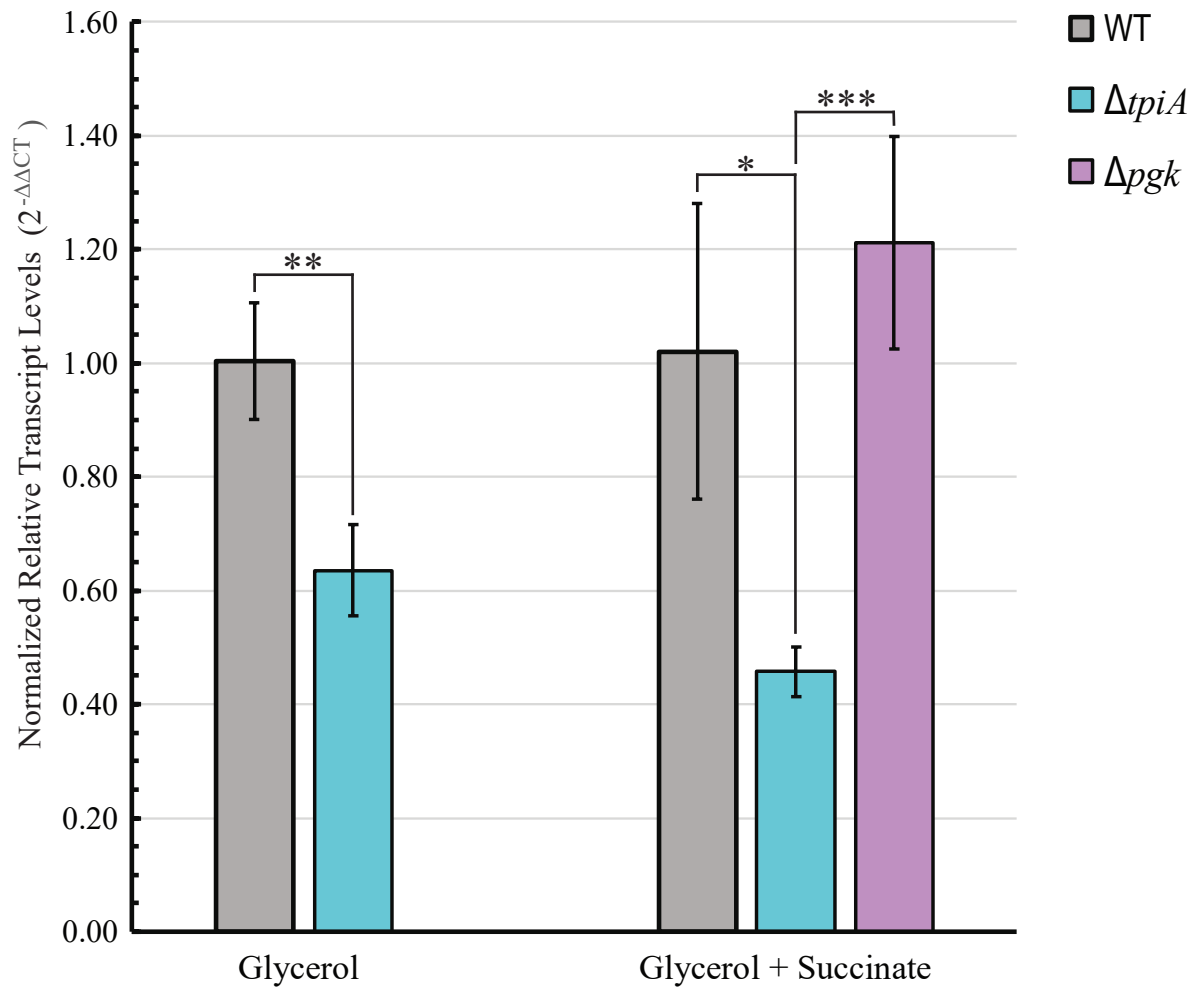

Supplement: Supplementary Materials — Supplementary Figures in GitLab: computationally identified EMP bypasses. Figure S1: growth of a Δtpi ΔmgsA strain on glycerol and succinate compared to a Δtpi strain. Figure S2: mgsA transcript levels determined by qPCR experiments. Figure S3: predicted and measured 13C-labeling in selected amino acids upon feeding of 1,6-13C2-glucose in cells using EMP-glycolysis, the methylglyoxal pathway, or the serine shunt. Figure S4: genome sequencing coverage of serine-tolerant Δeno isolates (G3 mutants). Figure S5: transcript level of serine shunt genes of the glycerol evolved iso1 strain. Figure S6: target specificity analysis of qPCR primers. Figure S7: serine-dependent growth of iso1 ΔserA strain compared to a WT-based ΔserA strain. Table S1: identified mutations different in the serine-tolerant Δeno strains compared to the reference strain. Table S2: identified mutations in the evolved Δ eno strains. Table S3: oligonucleotide primers used. Supplementary Method to the computational analysis to identify glycolytic bypasses in E. coli/A constraint-based method for finding glycolysis bypasses. Table S4. Allowed metabolite concentration ranges in the model. Table S5: RNA samples and reverse transcription information. [file 9859643.f1.zip › S2.pdf]

a

## EMP-pathway

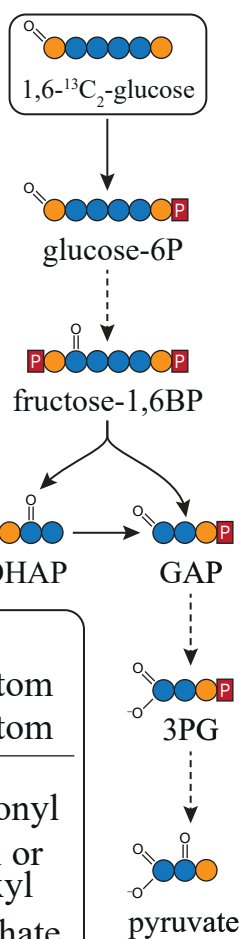

## MG-pathway

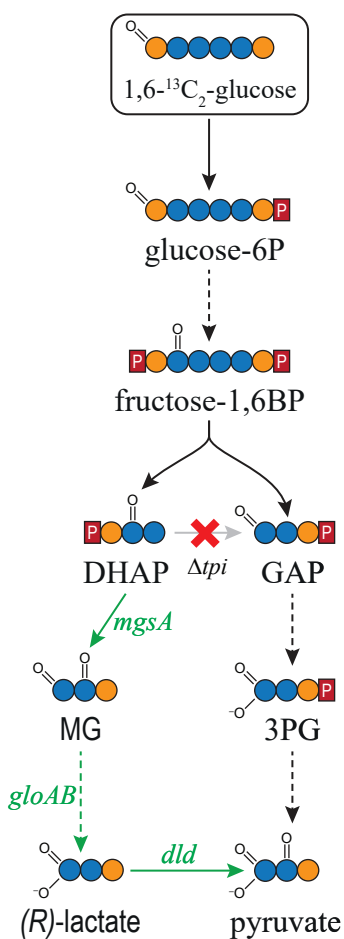

## Serine Shunt

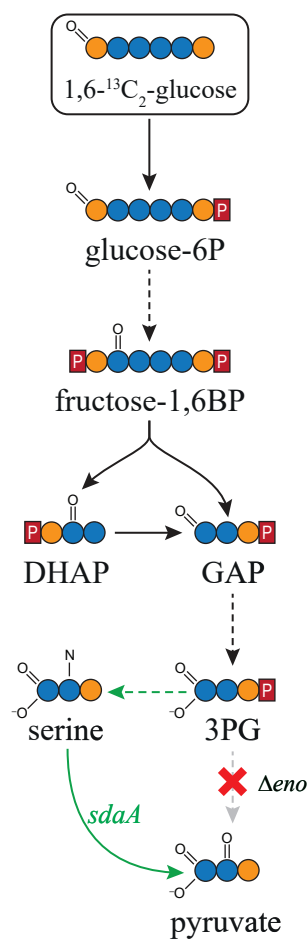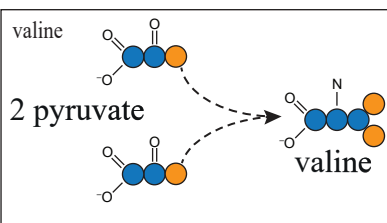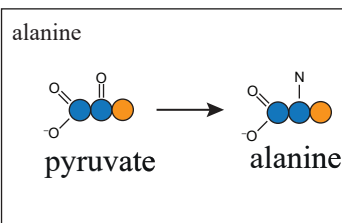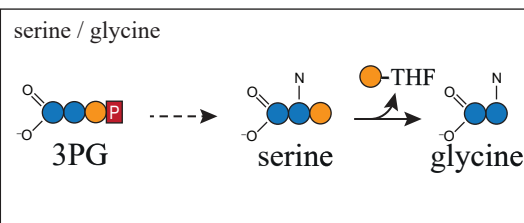

b

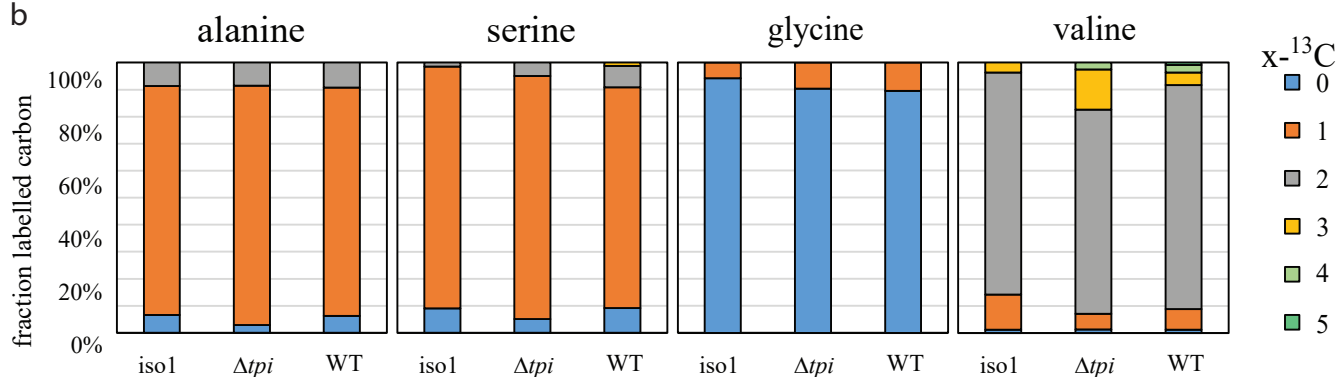

Supplement: Supplementary Materials — Supplementary Figures in GitLab: computationally identified EMP bypasses. Figure S1: growth of a Δtpi ΔmgsA strain on glycerol and succinate compared to a Δtpi strain. Figure S2: mgsA transcript levels determined by qPCR experiments. Figure S3: predicted and measured 13C-labeling in selected amino acids upon feeding of 1,6-13C2-glucose in cells using EMP-glycolysis, the methylglyoxal pathway, or the serine shunt. Figure S4: genome sequencing coverage of serine-tolerant Δeno isolates (G3 mutants). Figure S5: transcript level of serine shunt genes of the glycerol evolved iso1 strain. Figure S6: target specificity analysis of qPCR primers. Figure S7: serine-dependent growth of iso1 ΔserA strain compared to a WT-based ΔserA strain. Table S1: identified mutations different in the serine-tolerant Δeno strains compared to the reference strain. Table S2: identified mutations in the evolved Δ eno strains. Table S3: oligonucleotide primers used. Supplementary Method to the computational analysis to identify glycolytic bypasses in E. coli/A constraint-based method for finding glycolysis bypasses. Table S4. Allowed metabolite concentration ranges in the model. Table S5: RNA samples and reverse transcription information. [file 9859643.f1.zip › S3.pdf]

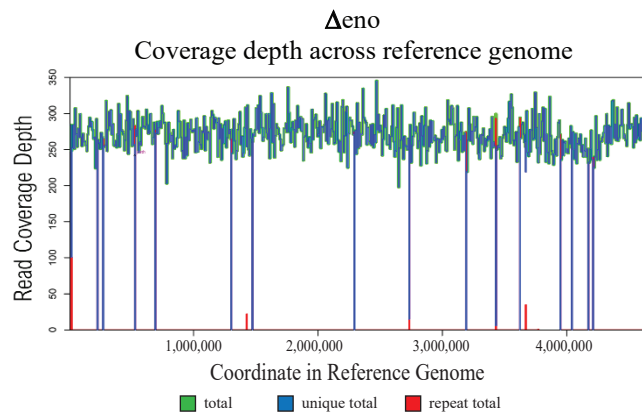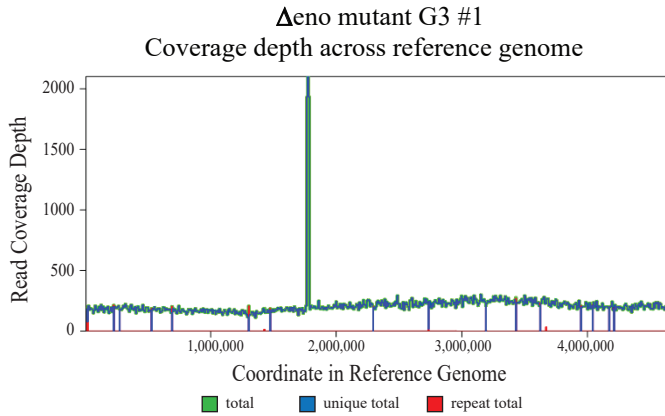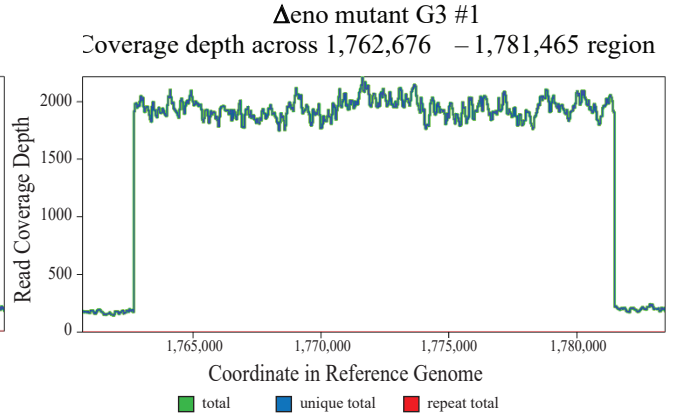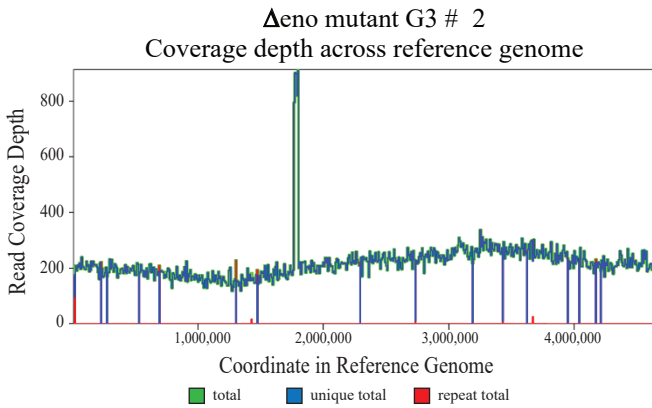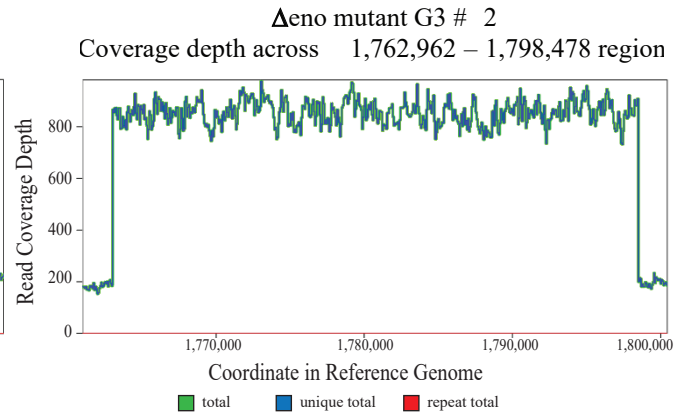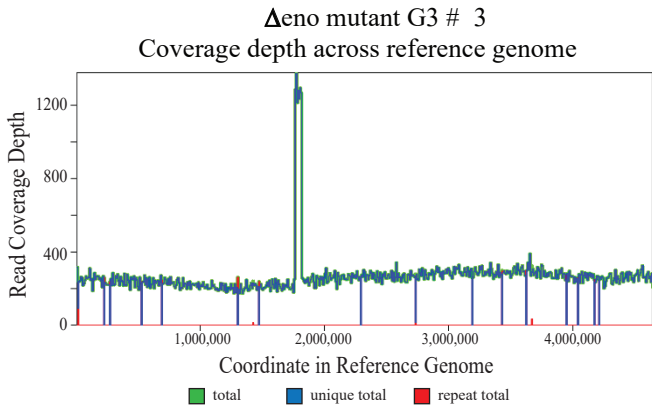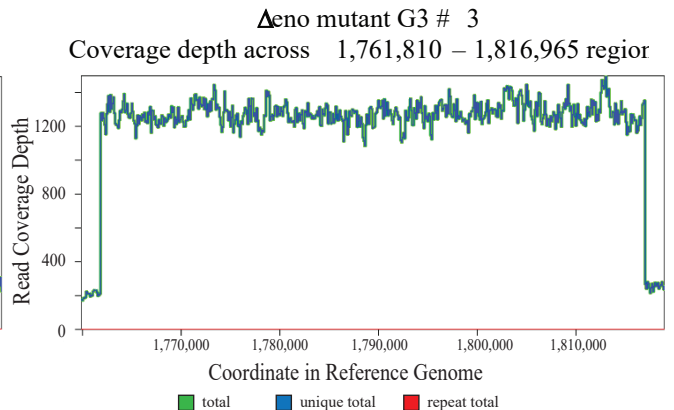

Supplement: Supplementary Materials — Supplementary Figures in GitLab: computationally identified EMP bypasses. Figure S1: growth of a Δtpi ΔmgsA strain on glycerol and succinate compared to a Δtpi strain. Figure S2: mgsA transcript levels determined by qPCR experiments. Figure S3: predicted and measured 13C-labeling in selected amino acids upon feeding of 1,6-13C2-glucose in cells using EMP-glycolysis, the methylglyoxal pathway, or the serine shunt. Figure S4: genome sequencing coverage of serine-tolerant Δeno isolates (G3 mutants). Figure S5: transcript level of serine shunt genes of the glycerol evolved iso1 strain. Figure S6: target specificity analysis of qPCR primers. Figure S7: serine-dependent growth of iso1 ΔserA strain compared to a WT-based ΔserA strain. Table S1: identified mutations different in the serine-tolerant Δeno strains compared to the reference strain. Table S2: identified mutations in the evolved Δ eno strains. Table S3: oligonucleotide primers used. Supplementary Method to the computational analysis to identify glycolytic bypasses in E. coli/A constraint-based method for finding glycolysis bypasses. Table S4. Allowed metabolite concentration ranges in the model. Table S5: RNA samples and reverse transcription information. [file 9859643.f1.zip › S4.pdf]

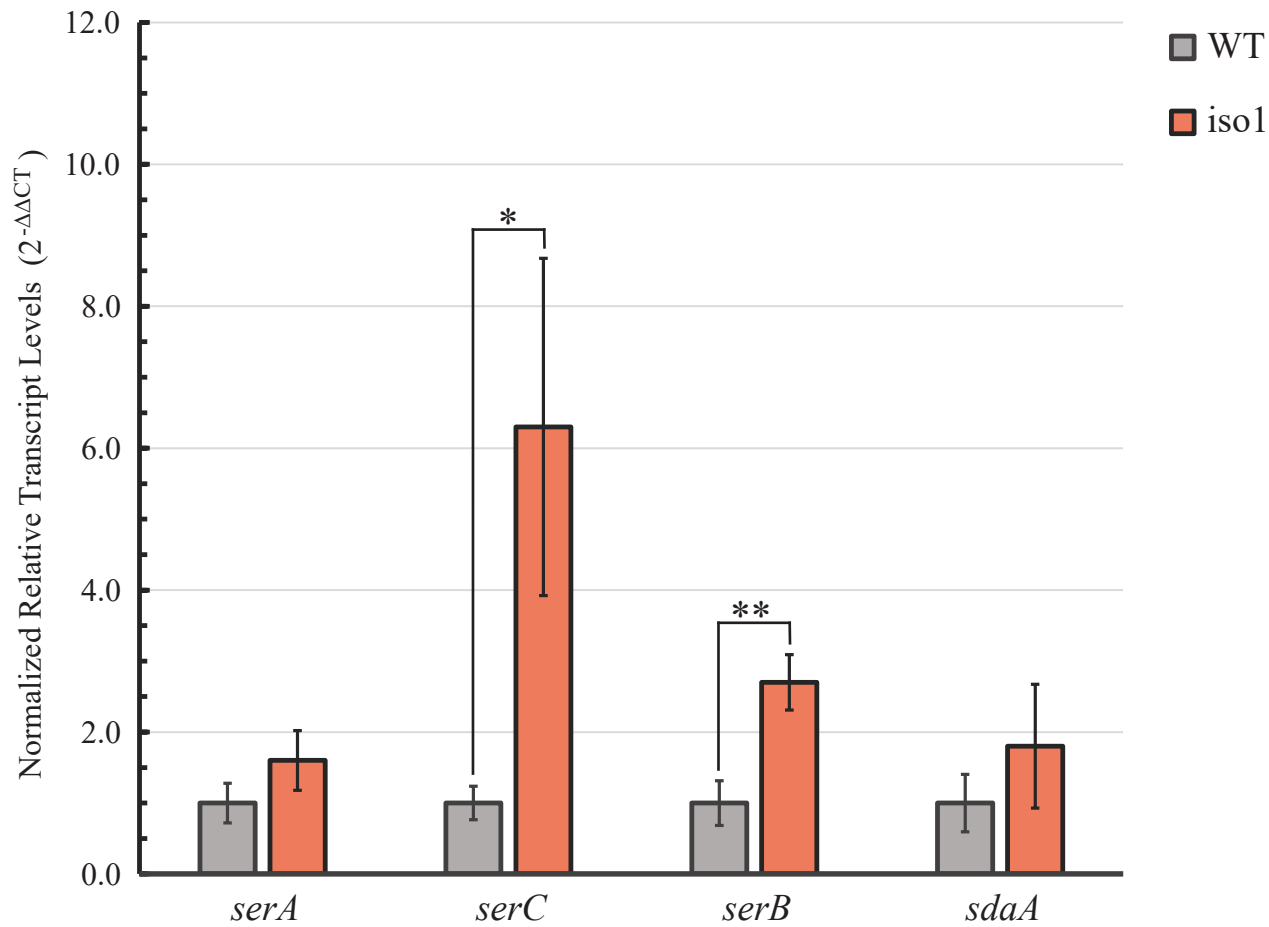

Supplement: Supplementary Materials — Supplementary Figures in GitLab: computationally identified EMP bypasses. Figure S1: growth of a Δtpi ΔmgsA strain on glycerol and succinate compared to a Δtpi strain. Figure S2: mgsA transcript levels determined by qPCR experiments. Figure S3: predicted and measured 13C-labeling in selected amino acids upon feeding of 1,6-13C2-glucose in cells using EMP-glycolysis, the methylglyoxal pathway, or the serine shunt. Figure S4: genome sequencing coverage of serine-tolerant Δeno isolates (G3 mutants). Figure S5: transcript level of serine shunt genes of the glycerol evolved iso1 strain. Figure S6: target specificity analysis of qPCR primers. Figure S7: serine-dependent growth of iso1 ΔserA strain compared to a WT-based ΔserA strain. Table S1: identified mutations different in the serine-tolerant Δeno strains compared to the reference strain. Table S2: identified mutations in the evolved Δ eno strains. Table S3: oligonucleotide primers used. Supplementary Method to the computational analysis to identify glycolytic bypasses in E. coli/A constraint-based method for finding glycolysis bypasses. Table S4. Allowed metabolite concentration ranges in the model. Table S5: RNA samples and reverse transcription information. [file 9859643.f1.zip › S5.pdf]

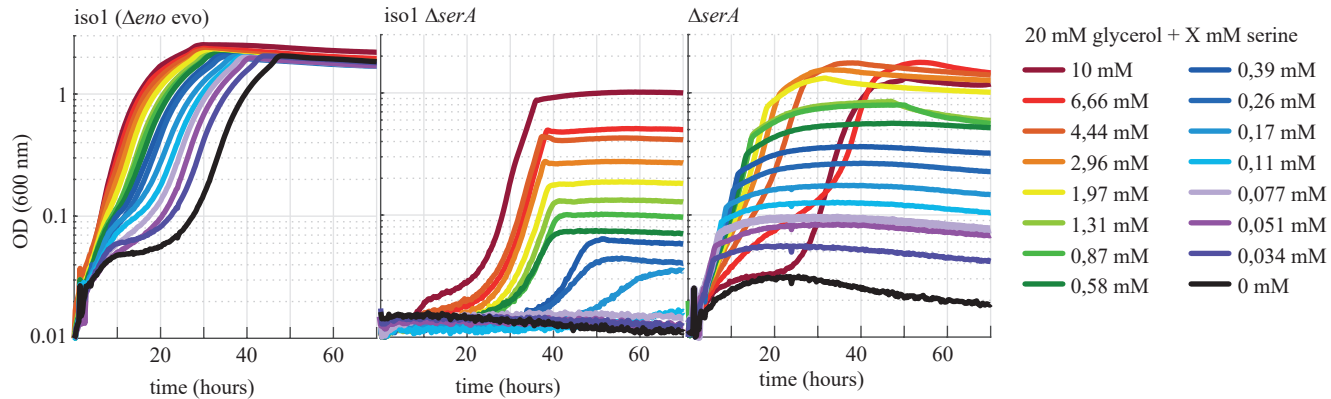

Supplement: Supplementary Materials — Supplementary Figures in GitLab: computationally identified EMP bypasses. Figure S1: growth of a Δtpi ΔmgsA strain on glycerol and succinate compared to a Δtpi strain. Figure S2: mgsA transcript levels determined by qPCR experiments. Figure S3: predicted and measured 13C-labeling in selected amino acids upon feeding of 1,6-13C2-glucose in cells using EMP-glycolysis, the methylglyoxal pathway, or the serine shunt. Figure S4: genome sequencing coverage of serine-tolerant Δeno isolates (G3 mutants). Figure S5: transcript level of serine shunt genes of the glycerol evolved iso1 strain. Figure S6: target specificity analysis of qPCR primers. Figure S7: serine-dependent growth of iso1 ΔserA strain compared to a WT-based ΔserA strain. Table S1: identified mutations different in the serine-tolerant Δeno strains compared to the reference strain. Table S2: identified mutations in the evolved Δ eno strains. Table S3: oligonucleotide primers used. Supplementary Method to the computational analysis to identify glycolytic bypasses in E. coli/A constraint-based method for finding glycolysis bypasses. Table S4. Allowed metabolite concentration ranges in the model. Table S5: RNA samples and reverse transcription information. [file 9859643.f1.zip › S7.pdf]
